# The expression characteristics and clinical significance of ACP6, a potential target of nitidine chloride, in hepatocellular carcinoma

**DOI:** 10.1186/s12885-022-10292-1

**Published:** 2022-12-01

**Authors:** Li Gao, Dan-Dan Xiong, Xia Yang, Jian-Di Li, Rong-Quan He, Zhi-Guang Huang, Ze-Feng Lai, Li-Min Liu, Jia-Yuan Luo, Xiu-Fang Du, Jiang-Hui Zeng, Ming-Fen Li, Sheng-Hua Li, Yi-Wu Dang, Gang Chen

**Affiliations:** 1 Department of Pathology, The First Affiliated Hospital of Guangxi Medical University, No.6 Shuangyong Rd, Guangxi Zhuang Autonomous Region, Nanning, 530021 People’s Republic of China; 2 Department of Medical Oncology, The First Affiliated Hospital of Guangxi Medical University, No.6 Shuangyong Rd, Guangxi Zhuang Autonomous Region, Nanning, 530021 People’s Republic of China; 3 Department of Pharmacy, Guangxi Medical University Cancer Hospital, No.71 Hedi Rd, Guangxi Zhuang Autonomous Region, Nanning, 530021 People’s Republic of China; 4 Department of Toxicology, College of Pharmacy, Guangxi Medical University, No.22 Shuangyong Rd, Guangxi Zhuang Autonomous Region, Nanning, 530021 People’s Republic of China; 5 Department of Clinical Laboratory, The Third Affiliated Hospital of Guangxi Medical University/Nanning Second People’s Hospital, No. 13 Dancun Road, Guangxi Zhuang Autonomous Region, Nanning, 530031 People’s Republic of China; 6 Laboratory Department, The First Affiliated Hospital of Guangzhou University of Traditional Chinese Medicine, No. 89-9 Dongge Road, Guangxi Zhuang Autonomous Region, Nanning, 530021 People’s Republic of China; 7 Department of Urology Surgery, The First Affiliated Hospital of Guangxi Medical University, No.6 Shuangyong Rd, Guangxi Zhuang Autonomous Region, Nanning, 530021 People’s Republic of China

**Keywords:** ACP6, HCC, NC, Xenograft, RNA-seq, Microarray

## Abstract

**Background:**

Acid phosphatase type 6 (ACP6) is a mitochondrial lipid phosphate phosphatase that played a role in regulating lipid metabolism and there is still blank in the clinico-pathological significance and functional roles of ACP6 in human cancers. No investigations have been conducted on ACP6 in hepatocellular carcinoma (HCC) up to date.

**Methods:**

Herein, we appraised the clinico-pathological significance of ACP6 in HCC via organizing expression profiles from globally multi-center microarrays and RNA-seq datasets. The molecular basis of ACP6 in HCC was explored through multidimensional analysis. We also carried out in vitro and in vivo experiment on nude mice to investigate the effect of knocking down ACP6 expression on biological functions of HCC cells, and to evaluate the expression variance of ACP6 in xenograft of HCC tissues before and after the treatment of NC.

**Results:**

ACP6 displayed significant overexpression in HCC samples (standard mean difference (SMD) = 0.69, 95% confidence interval (CI) = 0.56–0.83) and up-regulated ACP6 performed well in screening HCC samples from non-cancer liver samples. ACP6 expression was also remarkably correlated with clinical progression and worse overall survival of HCC patients. There were close links between ACP6 expression and immune cells including B cells, CD8 + T cells and naive CD4 + T cells. Co-expressed genes of ACP6 mainly participated in pathways including cytokine-cytokine receptor interaction, glucocorticoid receptor pathway and NABA proteoglycans. The proliferation and migration rate of HCC cells transfected with ACP6 siRNA was significantly suppressed compared with those transfected with negative control siRNA. ACP6 expression was significantly inhibited by nitidine chloride (NC) in xenograft HCC tissues.

**Conclusions:**

ACP6 expression may serve as novel clinical biomarker indicating the clinical development of HCC and ACP6 might be potential target of anti-cancer effect by NC in HCC.

**Supplementary Information:**

The online version contains supplementary material available at 10.1186/s12885-022-10292-1.

## Background

According to the statistics of latest epidemic research, liver cancer poses a serous threat to human health with high incidence rate and mortality rate ranking sixth and fourth worldwide, respectively [1, 2]. An overwhelming majority of liver cancer cases were hepatocellular carcinoma (HCC), which has a high prevalence in multiple countries [3, 4]. The risk factors for HCC are complex and vary from area to area. While non-alcoholic fatty liver disease, hepatitis C virus infection and alcohol abuse were considered as the main risk elements for HCC in Japan and Western countries; the culprit behind HCC in eastern Asia and sub-Saharan Africa is hepatitis B virus (HBV) [5–7]. Although great achievements have been made for extending the life expectancy of HCC patients and a combination of therapies including surgical removal, radiotherapy, chemotherapy, radiofrequency ablation (RFA), and molecular targeted therapy have been applied for the treatment of HCC patients, most HCC patients were found to be in middle or later stage when diagnosed and the five-year survival rate of HCC patients remained at low level [8–13]. For prolonged survival condition and improved life quality of HCC patients, it is imperative to further probe into the ecology and molecular mechanism of HCC and develop novel biomarkers for individual interventions.

Acid phosphatase type 6 (ACP6) is a mitochondrial lipid phosphate phosphatase that played a role in regulating lipid metabolism through hydrolyzation of lysophosphatidic acid to monoacylglycerol [14–16]. Two studies have revealed the down-regulation of ACP6 in esophageal squamous cell carcinoma and ovarian cancer as well as the impact of dys-regulated ACP6 on cancer progression [17, 18]. There is still blank in the clinico-pathological significance and functional roles of ACP6 in human cancers and no investigations have been conducted on ACP6 in HCC up to date.

In recent times, traditional Chinese medicine has gained popularity in the clinical management of human cancers due to its anti-tumor pharmacological action, synergism with chemotherapy and toxity-reducing effect [19, 20]. Nitidine chloride (NC) is a natural alkaloid extracted from the root of traditional Chinese herb Zanthoxylum nitidum and we have previously discovered the tumor-combating activities of NC in HCC [21–25]. NC exerted significant suppressive effect on HCC xenograft tumor growth and caused notable decrease in tumor size of NC-treated group compared with control group [22]. It would be interesting to analyze the influence of NC on expression profiles of genes in HCC.

Herein, we aimed to appraise the clinico-pathological significance of ACP6 in HCC via organizing expression profiles from globally multi-center microarrays and RNA-seq datasets. The molecular basis of ACP6 in HCC was explored through analysis of mutation landscape, distribution difference of expression at single cell level, correlation with immune cell infiltration and functional enrichment of co-expressed genes. We also carried out in vivo experiment on nude mice to evaluate the expression variance of ACP6 in xenograft of HCC tissues before and after the treatment of NC.

## Methods

### Comprehensive expression profiling analysis of ACP6 in HCC

#### Curation of public microarrays and RNA-seq datasets

RNA-seq datasets containing fragments per kilobase million (FPKM) gene expression matrix of HCC samples and non-cancer liver samples as well as the clinical information of these HCC patients were obtained from the cancer genome atlas (TCGA) database. To weaken the disparity in sample sizes of HCC and non-cancer liver tissues, we additionally downloaded transcripts per million (TPM) expression matrix of 50 non-cancer liver samples from the genotype-tissue expression project (GTEx) database. The FPKM gene expression matrix of 371 HCC samples and 226 non-cancer liver samples were converted into the data format of TPM gene expression matrix and combined with the TPM expression matrix of 50 non-cancer liver tissues from GTEx project into a single log2 transformed RNA-seq dataset. Microarrays that included expression data of ACP6 in no less than three human HCC and non-cancer liver specimen were collected from gene expression omnibus (GEO) and ArrayExpress databases. All microarrays were normalized with limma package in R software for correcting the heterogeneity between HCC and non-cancer cases. Average expression values were calculated for genes with duplicated probe IDs and only one record was reserved for each gene. For processed microarrays from the same platform, batch effect between these microarrays was removed with sva package in R software v.4.0.1 and these microarrays were integrated into a single large microarray set.

#### Differential expression analysis

Violin plots and receiver’s operating characteristics (ROC) curves that reflected the expression pattern of ACP6 in HCC and non-cancer liver specimen were plotted for each included RNA-seq dataset and microarrays with the pROC, ggplot2 and patchwork packages in R software v.3.6.1. Expression data of ACP6 in HCC and non-cancer liver samples were extracted from the above sorted RNA-seq dataset and microarrays, based on which standard mean difference (SMD) forest plot and summarized receiver’s operating characteristics (SROC) curves were created through meta package in R software v.3.6.1 and Stata v.14.0 for overall evaluation of the differential expression of ACP6 in HCC and non-cancer liver samples as well as the capacity of abnormal ACP6 expression in discerning HCC from non-cancer liver tissues.

### The effect of ACP6 expression on the overall survival of HCC patients

Firstly, we searched in GEO, ArrayExpress and other literature databases to seek datasets with both prognostic information of HCC patients and expression data of ACP6 in HCC cases. Univariate cox regression analysis and Kaplan–Meier survival analysis were carried out on all prognostic dataset with ggplot2, survminer and survival packages in R software. HCC patients were grouped according to the median value of ACP6 expression and *p* < 0.05 from log-rank test indicated significant different survival between HCC patients with low or high ACP6 expression. A series of hazard ratio (HR) values with 95% confidence interval (CI) generated from univariate cox regression analysis were arranged as data input of aggregation by meta package in R software. The effect of ACP6 expression on the overall survival probability of HCC patients was estimated by pooled HR values in forest plot.

### Genetic alteration status of ACP6 in HCC patients

We referred to cBioPortal database to check the mutation types and z-scores of mRNA expression (log RNA Seq V2 RSEM) of ACP6 in 440 HCC samples. The genomics data for Oncoprint module in cBioPortal database were provided by GDAC Firehose project.

Relationship between ACP6 expression and infiltration level of immune cells in HCC.

The proportions of 22 immune and stromal cells such as B cells naive, B cells memory, Plasma cells, T cells CD8 and T cells CD4 naive in dataset covering most HCC samples were reckoned from expression matrix with the methods of CIBERSORT in R software v.4.0.1. The different proportions of these immune or stromal cells in HCC patient groups divided by median ACP6 expression value was analysed by independent student’s t test and visualized as box plot in GraphpadPrism v.8.0.1.

### Expression analysis of ACP6 in liver-resident immune cells at single cell level

The expression abundance difference of ACP6 in vairous clusters of liver-resident immune cells was analysed through methods of t-distributed stochastic neighbor embedding (tSNE) in Single Cell Expression Atlas. A total of 61,144 cells were involved in single-cell RNA-sequencing and the distribution of ACP6 expression values in different clusters of liver-resident immune cells were shown as tSNE plots.

### Construction of co-expression network for ACP6 in HCC

Multiscale Embedded Gene Co-expression Network Analysis (MEGENA) was performed on microarray with most HCC cases for identifying modules of co-expressed genes in HCC. Differential expression analysis by limma package in R software was conducted before MEGENA with the purpose of narrowing down the scope of genes involved in construction of co-expression network (log2FC > 0.5&log2FC < -0.5, adj.*p* < 0.05). The codes of executing MEGENA using MEGANA package in R software v.4.0.1 in the present study was attached in Additional file 1. Genes that clustered in the same module with ACP6 were regarded as co-expressed genes of ACP6 in HCC. Terms of enriched biological processes and pathways were annotated for co-expressed genes of ACP6 via Metascape. Adjusted *P* value < 0.05 demonstrated significant enrichment.

### In vitro experiments

#### Cell lines

The human HCC cell lines Huh7 were purchased from Procell Life Science&Technology Co.,Ltd. (Wuhan, China) and cultured in Dulbecco’s modified eagle’s medium (DMEM) (Gibco) supplemented with 10% fetal bovine serum (FBS), 100 U/mL penicillin G, and 100 g/ml streptomycin. All cells were maintained in a humidified incubator at 37℃ under the atmosphere of 5% CO_2_.

#### siRNA transfection

ACP6 siRNAs and negative control (NC) RNAs used for this study was designed and synthesized by Sangon Biotechnology Co., Ltd (Shanghai). The sequences of ACP6 siRNAs and the NC RNAs were as follows: GCAGAUUCAGAAGUCUUGUAUTT (ACP6 siRNA sense), AUACAAGACUUCUGAAUCUGCTT (ACP6 siRNA anti-sense), UUCUCCGAACGUGUCACGUTT (NC sense), ACGUGACACGUUCGGAGAATT (NC anti-sense). Before the transfection experiment, the cells were seeded on the cell plate and added with DMEM complete medium to make the confluence of cells reach 60–90% within 24 h. The cells were transfected with ACP6 siRNA and NC RNA at the concentration of 10 nM using RNATransMate according to the the manufacturer’s protocol. After transfection for 24 h, the medium was replaced with DMEM complete medium.

#### RT-qPCR

Total RNA in Huh7 cells transfected with ACP6 siRNA and NC RNA was extracted through Tiangen RNAsimple total RNA kit (DP419, TIANGEN Biotech CO., LTD, Beijing China) according to the kit instructions. The concentration of extracted total RNA was determined by NanoDrop 2000. PrimeScript™ RT reagent Kit with gDNA Eraser (Perfect Real Time) (RR047A, Takara Bio) in a total volume of 20 μl and ChamQ Universal SYBR qPCR Master Mix (2x) in a total volume of 20 µL were used in the present study for conducting real-time quantitative PCR with the Bio-Rad CFX96 system. The sequences of ACP6 and GAPDH primers were: 5’-GATTTGCAAGGATGATCGAACA-3’ (ACP6, forward) (Sangon Biotechnology Co., Ltd, Shanghai), 5’-CATCTGAAGACTTTCCCTGTCT-3’ (ACP6 reverse) (Sangon Biotechnology Co., Ltd, Shanghai), 5’-GCACCGTCAAGGCTGAGAAC-3’ (GAPDH, forward) (Takara Bio), 5’- TGGTGAAGACGCCAGTGGA-3’) (GAPDH reverse) (Takara Bio). The expression value of ACP6 in Huh7 cells was calculated by the 2-ΔΔCt method.

#### CCK8 cell proliferation assay

We performed CCK8 assay to detect the influence of knocking down ACP6 on proliferation of Huh7 cells. Firstly, Huh7 cells (5 × 10^3^) were seeded into 96-well plates with 100 µL DMEM complete medium per well for 24. Each group contained five duplicate wells. Then, 1 pmol ACP6 siRNA and NC RNA were added into the wells and the cells were incubated for 24 h before replacement of medium. CCK8 reagent (10 µL) (CK04, DOJINDO) was mixed with the medium at 24, 48 and 72 h for 2 h under dark conditions at 37℃. The OD values of each well at a wavelength of 450 nm were measured by a multi-well plate reader.

#### Scratch assay

Monolayer ACP6 siRNA and NC RNA transfected Huh7 cells with a confluence of 90% in 6-well plate was wounded with 200 µL pipette tips. After removal of floating cells by PBS washing for two times, the cells were incubated with serum-free DMEM for 48 h. The migration of cells was observed with microscope and three randomly selected fields were photographed for each well at 0 and 48 h. The percentage of wound healing area was calculated through Image J software (the remaining area at 48 h / the remaining area at 0 h).

### The expression change of ACP6 in HCC before and after the treatment of NC

Traditional Chinese medicine is one of the hotspots in the fields of cancer research. Out of curiosity, we also performed in vivo experiments to investigate the influence of NC on the expression of ACP6 in HCC. Nude mice used for in vivo experiments were purchased from Guangxi Medical University Animal Center (Guangxi, China). The detailed steps of in vivo experiments on nude mice were stated in previous work [22]. BALB/c nude mice obtained from Guangxi Medical University Animal Center (Guangxi, China) were incubated under pathogen-free conditions at room temperature. Injection of SMMC7721 cells at a concentration of 1 × 107 cells/L was performed on the right armpit of each mouse. After fourteen days of raising, mice with obvious formation of xenograft (70 mm3) were randomly divided into negative control group and the NC group. Saline and 7 mg/kg/d NC were given to mice in negative control group and the NC group, respectively. Drug administration was conducted at the frequency of every other day, the mice were euthanized after 15 days and the tumor tissues treated with saline or nitidine chloride were reaped for extraction of total RNA. Then mRNA-sequencing was conducted following the procedures of NEB Next® UltraTM Directional RNA Library Prep Kit for Illumina® (NEB, USA) [22]. Significant differentially expressed genes between control or NC-treated HCC xenograft tissues were selected using DESeq2 package in R software v.3.3.2 (|log2FC|> 1, adj.*p* < 0.05). The in vivo experiments in the current study adhered to the ethical standards proposed by Guide for the Care and Use of Laboratory Animals (the Shanghai SLAC Laboratory Animal of China, 2015) and were approved by the Ethics Committee of the First Affiliated Hospital of Guangxi Medical University.

### Statistical analysis

Expression value of ACP6 in alll included RNA-seq dataset and microarrays were presented as mean (M) ± standard deviation (SD). The associations between ACP6 expression and clinico-pathological parameters of HCC patients from the cohort of RNA-seq dataset were analysed with independent student’s t test or analysis of variance in SPSS v.22.0. Statistical significance was indicated by *p* < 0.05.

## Results

### The significant aberrant expression of ACP6 between HCC and non-cancer liver samples

TCGA-GTEx RNA-seq dataset and 38 microarrays processed after removal of batch effects were enrolled for differential expression analysis in the present work (Additional file 2). The procedures of selecting appropriate RNS-seq dataset and microarrays for the current study were exhibited in Additional file 3. It could be seen from the violin plots and ROC curves that ACP6 expression was obviously higher in HCC than in non-cancer liver specimen in most RNA-seq dataset and microarrays and overexpressed ACP6 showed preferable performance in distinguishing HCC and non-cancer liver samples (Additional files 4 and 5). The overall forest plot of SMD and SROC curves generated through pooling expression data of ACP6 in HCC and non-cancer liver samples from all included RNA-seq dataset and microarrays confirmed the significant overexpression of ACP6 in HCC tissues and the preferable discriminatory ability of ACP6 expression for HCC (SMD = 0.69, 95%CI = 0.56–0.83) (AUC = 0.71, 95%CI = 0.67–0.75) (Additional file 6). With regard to the relationships between ACP6 expression and the clinico-pathological features of HCC patients, up-regulation of ACP6 was remarkably associated with mild adjacent hepatic tissue inflammation, moderate fibrosis ishak score, history of hepatitis B and higher histologic grade (*p* < 0.05) (Additional file 7).

### The effect of ACP6 expression on the overall survival of HCC patients

The prognostic value of ACP6 expression for predicting overall survival outcome of HCC patients was analysed in three cohorts from E-TABM-36, GSE76427 and TCGA database. The results from univariate cox regression analysis were listed in Table 1. Although the overall survival difference between HCC patients with high or low ACP6 expression reflected by Kaplan–Meier survival curves were insignificant. Forest plot of HR values suggested the trend that higher ACP6 expression might serve as a risk factor for the overall survival of HCC patients (HR = 1.29, 1.01–1.66) (Additional file 8).

**Table 1:** Analysis results from univariate cox regression analysis

Dataset ID	Hazard ratio	Lower limit	Upper limit	*P* value	Survival type
E-TABM-36	1.830072711	0.614994631	5.44584613	0.277383588	Overall survival
GSE76427	0.810388331	0.346374126	1.896011276	0.627827336	Overall survival
TCGA	1.32555326	1.012956906	1.734616188	0.039997151	Overall survival

### Genetic alteration status of ACP6 in HCC patients

As shown in the bar chart of Additional file 9, 14% of sequenced HCC tissues from GDAC Firehose project bore genetic alterations. Among the HCC cases with genetic mutations, amplification and mRNA high dominated the type of genetic mutations (Additional file 9).

### Relationship between ACP6 expression and infiltration level of immune cells in HCC

**Fig. 1:**
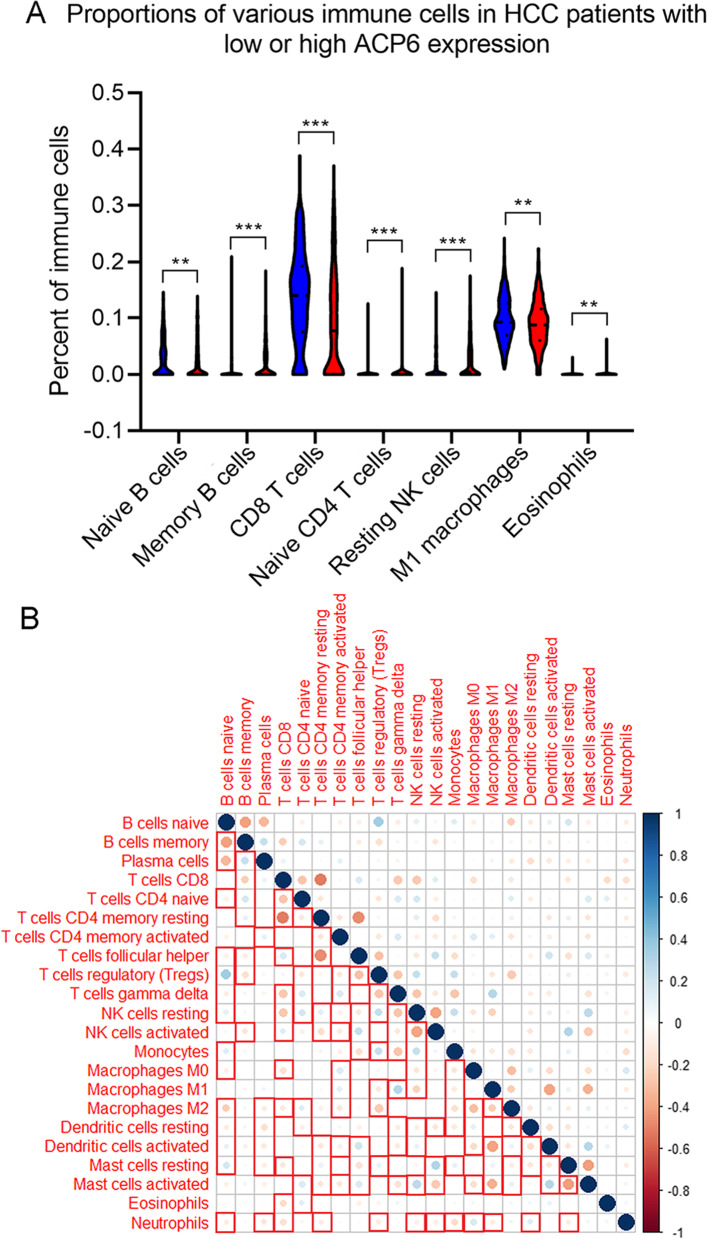
Infiltration levels of diverse immune cells in HCC patients with low or high ACP6 expression. **A** Violin plots of percents of various immune cells. **B** Correlation plot of infiltration levels of different immune cells. Significant correlation pairs were marked in red

GPL570 was chosen for immune correlation analysis because it included the largest HCC samples among all included datasets. Memory B cells, naive CD4 T cells, resting memor CD4 T cells, resting NK cells, monocytes, M2 macrophages, resting mast cells, activated mast cells and eosinophils infiltrated at higher levels in HCC patients with higher ACP6 expression while naive B cells, CD8 T cells, M1 macrophages and resting dendritic cells were found have a higher proportion in HCC patients with lower ACP6 expression (*p* < 0.05) (Table 2). The violin plot in Fig. 1A displayed part of the significant distinct distributions of immune cells in HCC patients with low or high ACP6 expression. There were significant inter-correlations between the infiltration levels of various immune cells including NK cells, B cells and CD8 + T cells in HCC samples from GPL570 (Fig. 1B).

**Table 2:** The relationship between ACP6 expression and the proportions of various immune cells in HCC

Types of immune cells	Expression group	Proportions of immune cells in low or high ACP6 expression groups				
		Number	Mean	Standard deviation	t	*P*-value
Naive B cells					-3.192	0.001
	ACP6 low expression	422	0.03	0.035		
	ACP6 high expression	422	0.022	0.034		
Memory B cells					5.108	< 0.001
	ACP6 low expression	422	0.011	0.024		
	ACP6 high expression	422	0.021	0.033		
CD8 T cells					-7.876	< 0.001
	ACP6 low expression	422	0.138	0.084		
	ACP6 high expression	422	0.091	0.089		
Naive CD4 T cells					5.121	< 0.001
	ACP6 low expression	422	0.002	0.01		
	ACP6 high expression	422	0.009	0.027		
Resting memory CD4 T cells					2.327	0.02
	ACP6 low expression	422	0.093	0.085		
	ACP6 high expression	422	0.107	0.098		
Resting NK cells					4.438	< 0.001
	ACP6 low expression	422	0.016	0.027		
	ACP6 high expression	422	0.025	0.036		
Monocytes					2.472	0.014
	ACP6 low expression	422	0.026	0.025		
	ACP6 high expression	422	0.03	0.028		
M1 macrophages					-3.025	0.003
	ACP6 low expression	422	0.098	0.039		
	ACP6 high expression	422	0.09	0.042		
M2 macrophages					2.365	0.018
	ACP6 low expression	422	0.143	0.062		
	ACP6 high expression	422	0.154	0.073		
Resting dendritic cells					-2.328	0.02
	ACP6 low expression	422	0.052	0.045		
	ACP6 high expression	422	0.045	0.048		
Resting mast cells					2.25	0.025
	ACP6 low expression	422	0.014	0.022		
	ACP6 high expression	422	0.018	0.031		
Activated mast cells					2.38	0.018
	ACP6 low expression	422	0.03	0.034		
	ACP6 high expression	422	0.036	0.041		
Eosinophils					2.716	0.007
	ACP6 low expression	422	0.001	0.004		
	ACP6 high expression	422	0.002	0.007

### Expression analysis of ACP6 in liver-resident immune cells at single cell level

**Fig. 2:**
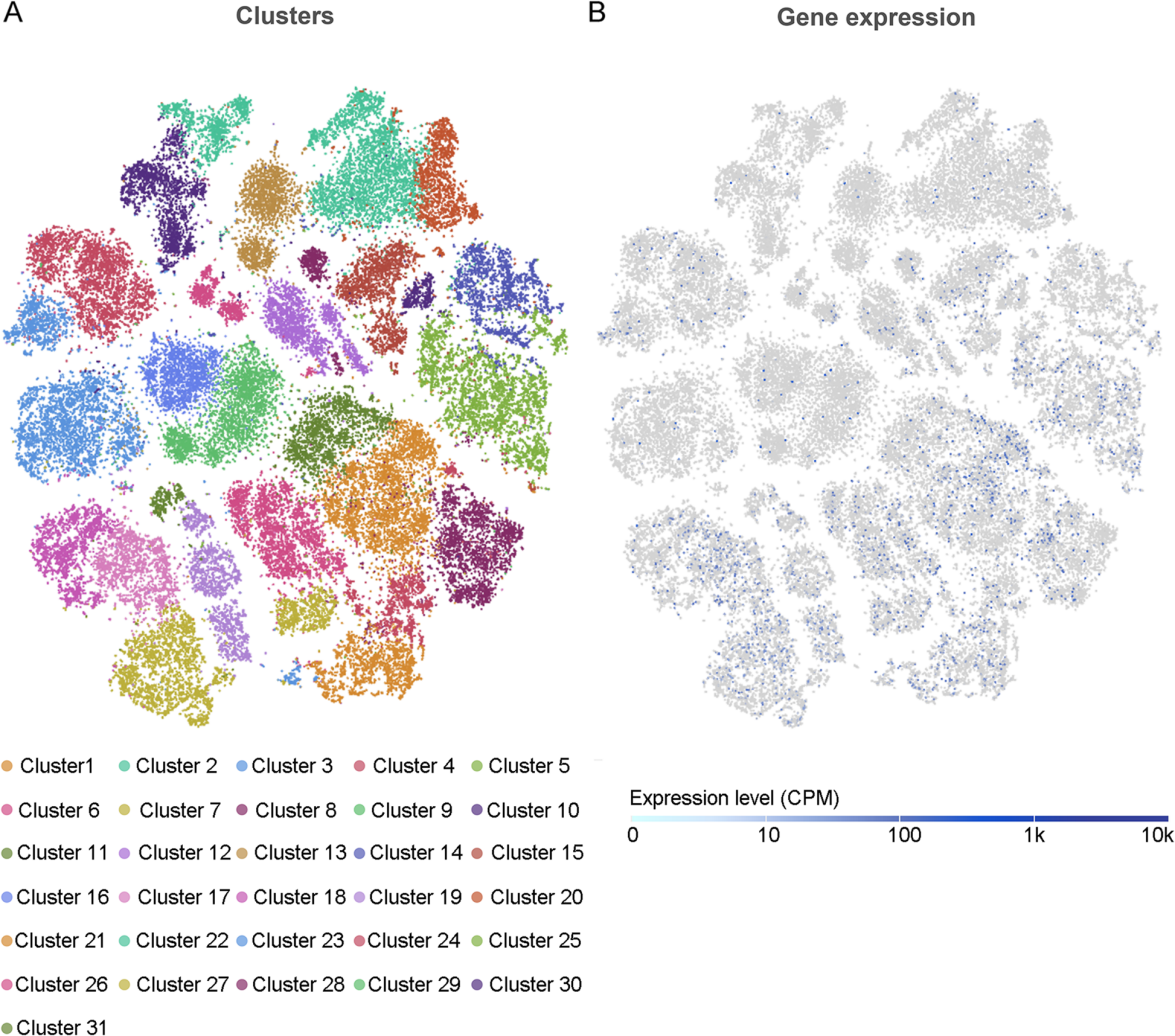
Distribution of ACP6 expression in different clusters of liver-resident immune cells at single cell level. **A** t-distributed stochastic neighbor embedding plot of various clusters of liver-resident immune cells. **B** t-distributed stochastic neighbor embedding plot of ACP6 expression level in different clusters. CPM: count per million

Liver-resident immune cells in human were grouped into 31 clusters by single cell sRNA sequencing and ACP6 expression mainly enriched in cluster 1, cluster7 and cluster 17 (Fig. 2).

### Construction of co-expression network for ACP6 in HCC

**Fig. 3:**
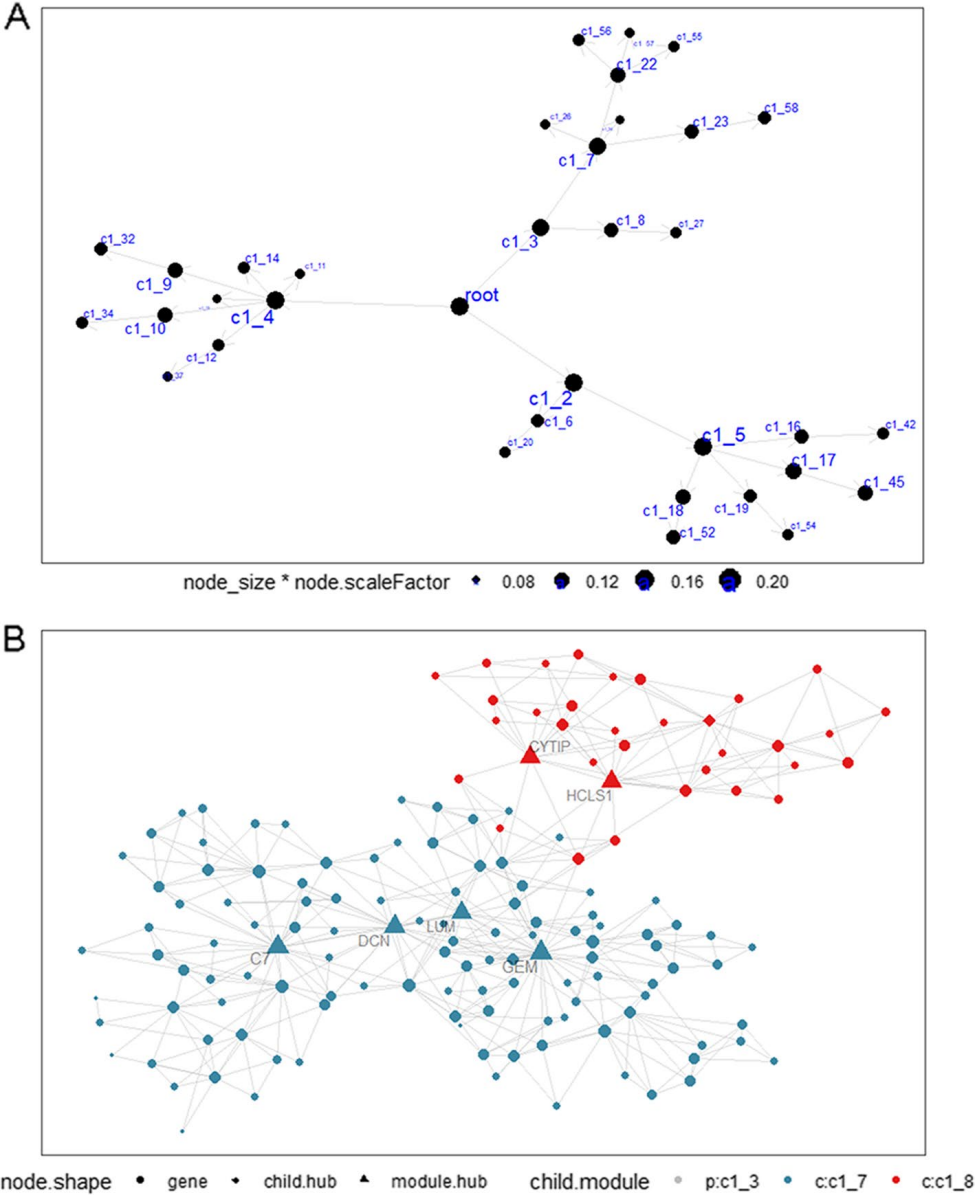
MEGENA analysis results for differentially expressed genes and ACP6 in GPL570. **A** Hierarchy network of all co-expressed modules. **B** The interactions between genes in c1_3 module. Hub genes were highlighted in triangles

For the efficient operation of MEGENA analysis, 1198 genes that presented upregulation or downregulation (logFC > 0.5 or < -0.5 and adj.*p* < 0.05) in HCC cases as well as ACP6 from GPL570 dataset were included for construction of co-expression network. A total of 58 co-expressed modules were identified by MEGENA algorithm and 150 genes were co-expressed with ACP6 in c1_3 module. Particularly six genes including C7, DCN, LUM, GEM, CYTIP and HCLS1 were hub genes of c1_3 module (Fig. 3). Genes co-expressed with ACP6 in GPL570 dataset were active in biological processes and pathways such as angiogenesis, cytokine-cytokine receptor interaction and extracellular matrix organization (Additional file 10).

### The influence of knocking down ACP6 expression on the proliferation and migration of HCC cells

**Fig. 4:**
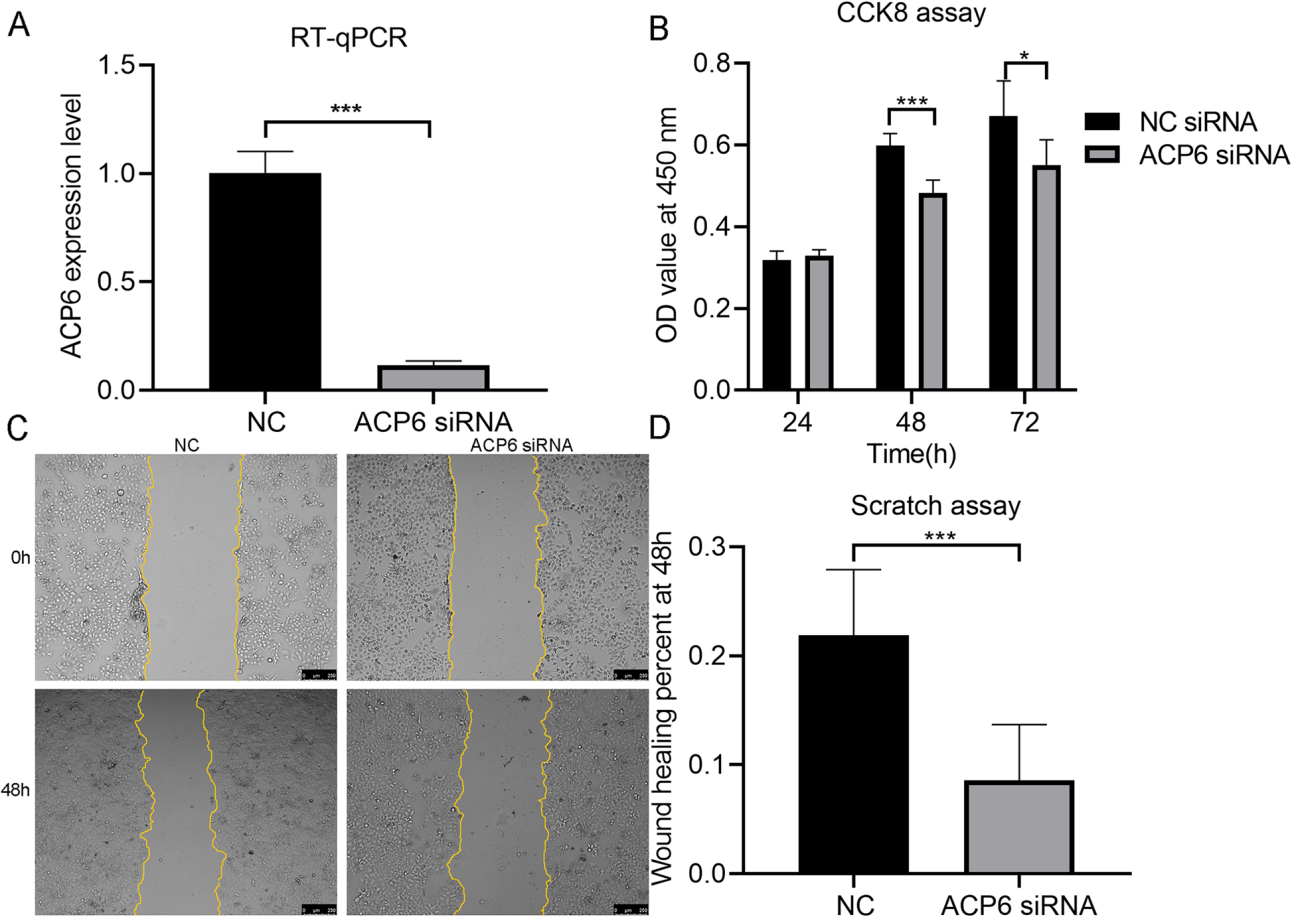
The influence of knocking down ACP6 expression on biological functions of Huh7 cells. **A** Expression of ACP6 mRNA between Huh7 cells transfected with negative control (NC) RNA and ACP6 siRNA. **B** Box plot of OD values from CCK8 assay. **C** Images of cell migration around scratches. **D** Box plot of wound healing percentage at 48 h. *: *P* < 0.05; ***: *P* < 0.001

Compared with Huh7 cells transfected with NC RNA, the expression level of ACP6 in Huh7 cells transfected with ACP6 siRNA was significantly decreased (1.003 ± 0.099; 0.116 ± 0.020) (*P* < 0.001) (Fig. 4A). The proliferative ability of Huh7 cells was remarkably inhibited by ACP6 siRNA at 48 h and 72 h (*P* < 0.05) compared with the group transfected with NC RNA (Fig. 4B). There were less Huh7 cells that migrated across the scratch area at 48 h in the group of transfection with ACP6 siRNA than the group of transfection with NC RNA (*P* < 0.001) (Fig. 4C and D).

### The expression change of ACP6 in HCC before and after the treatment of NC

**Fig. 5:**
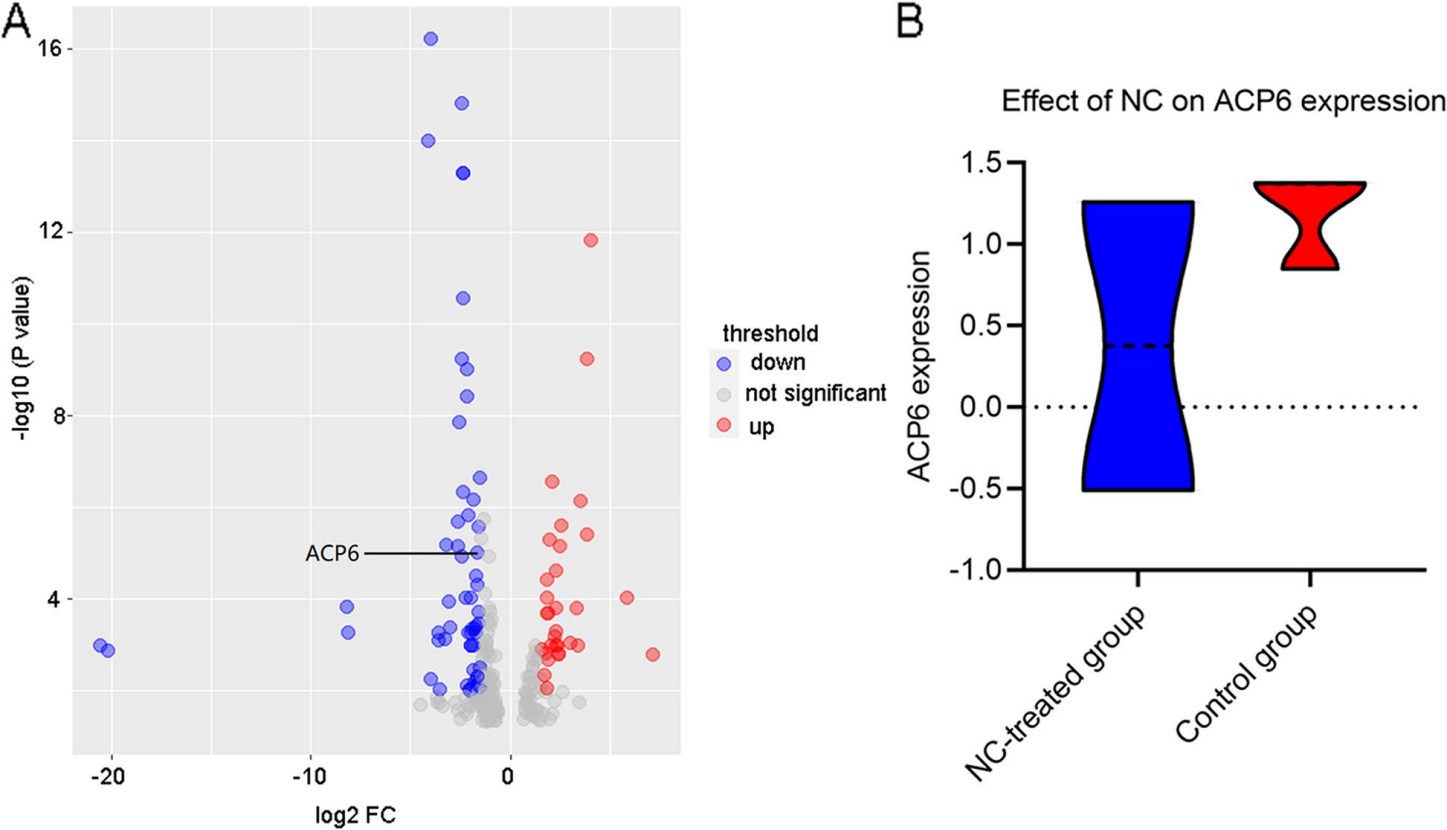
The effect of NC on ACP6 expression in HCC xenograft tissues. **A** Volcano plot of differentially expressed genes after the treatment of NC. **B** Violin plot of ACP6 expression in HCC xenograft tissues before and after the treatment of NC

Because the tumorigenesis effect of Huh7 cells on nude mice was poor, other cell lines were subcutaneously injected into the nude mice and SMMC7721 cells were found to have the best effect of tumor implantations. Therefore, SMMC7721 cell line was used for mice tumor implantation experiments. After quality control by principal component analysis, one NC-treated sample was excluded as the outlier and two NC-treated and three control HCC xenograft tumor tissues were judged as qualified for expression analysis of mRNA. ACP6 was among the 137 mRNAs that exhibited significant down-expression after the treatment of NC (log2FC = -1.661, adj.*p* < 0.001) (Fig. 5A). ACP6 expression was obviously lower in NC-treated group than in control group (Fig. 5B).

## Discussion

To our knowledge, the current study is the first to investigate the clinico-pathological significance and molecular basis of ACP6 in HCC through comprehensive analysis of global multi-center RNA-seq datasets and microarrays.

Before in-depth elaboration of the role of ACP6 in the carcinogenesis of HCC, it was important to evaluate the differential expression and clinico-pathological significance of ACP6 in HCC. The superiority of the present study over previous single gene research in cancer lied in the large samples and advanced algorithm of integration through calculating SMD values and plotting SROC curves. The 3857 HCC samples and 3132 non-cancer liver tissues involved for expression analysis in the present study were compiled from all public RNA-seq dataset and microarrays pertaining to expression matrix of HCC and non-cancer liver samples worldwide, which adheres to the concept of evidence-based medicine. For reducing systematic errors and properly assessing the differential expression of ACP6 between HCC and non-cancer liver tissues, we rigorously processed all included datasets through removal of batch effect and normalization. SMD values and SROC curves were further deducted from expression values of ACP6 in all included datasets, which guaranteed the credibility of results in the current work. Unlike the down-regulation and anti-carcinogenic effect of ACP6 reported in esophageal squamous cell carcinoma and ovarian cancer [17, 18], overexpression of ACP6 in HCC and the positive relationships between ACP6 expression and clinical indicators of the progression of HCC discovered from the present study implied that ACP6 might play diverse roles in different human cancers. In addition to the above analysis, we also evaluated the prognostic stratification capacity of ACP6 expression for HCC patients in multiple datasets. Although Kaplan–Meier survival curves revealed no significant survival difference between HCC patients with low or high ACP6 expression, the conclusion results from forest plot of HR values supported that ACP6 overexpression represents a hazard to the overall survival of HCC patients.

After acquiring general impressions of the expression pattern and clinico-pathological significance of ACP6 in HCC, we digged into the molecular basis of ACP6 in the formation and development of HCC to pave the way for clinical translational research on ACP6 in HCC. Firstly, we explored the profound reasons behind the overexpression of ACP6 in HCC at transcription level. It could be inferred from the large percentage of amplification and mRNA high among all mutation types that the upregulation of ACP6 in HCC might be attributed to genetic alterations such as amplification and mRNA high occurring in the genome of ACP6. Because tumor microenvironment played essential roles in regulating various biological events of human cancers, we analysed the correlation between ACP6 expression and infiltration level of a wide range of immune cells in HCC. The results from immune correlation analysis implied that the inclination of infiltration of several types of immune cells was closely linked with ACP6 expression in HCC patients. Specific distribution of ACP6 expression in certain clusters of liver-resident immune cells tuned with the analysis results from CIBERSORT methods, from which we speculated that ACP6 expression in HCC might be intervened by some components of tumor microenvironment in HCC; alternatively, the abnormal expression of ACP6 in HCC might affect immune cells such as memory B cells, naive CD4 T cells, resting memory CD4 T cells and resting NK cells, thus reshaping the tumor microenvironment of HCC. Except for ACP6 itself, genes that co-expressed with ACP6 were also worthy of attention, thus the research ideas of the current work were not confined to ACP6 merely. Through functional enrichment analysis of genes co-expressed with ACP6, a string of biological processes and pathways with implications of the action mechanisms of ACP6 in the oncogenesis of HCC were screened. Nearly half of the significant biological processes and pathway terms were intimately linked with the initiation and development of a wide type of human cancers [26–34]. Specifically, the contribution of angiogenesis, MAPK cascade and humoral immune response to the malignant process of HCC has been recorded in previous literature studies [35–37]. The serological testing and immunoblot assay by M Volkmann et al. revealed humoral immune response to p53 exclusively in HCC patients and the presence of p53 antibodies in HCC patients was not dependent on alpha-fetoprotein level [35]. The mitogen-activated protein kinase (MAPK) signaling pathways played essential roles in diverse biological events including survival, dissemination, and resistance to drug therapy of human tumor cells [38–40]. Wang et al. reported activation of MAPK signaling pathway during the promotion of HCC development stimulated by linc00601 upregulation [36]. HCC is rich in blood supply, and angiogenesis was indispensable for tumor growth, invasion and metastasis [41]. The work of Wang et al. disclosed that morphine could induce angiogenesis in HCC through activating PI3K/Akt/HIF-1α pathway and up-regulating VEGF expression [37]. Despite there have been no studies on the parts of ACP6 in activities of the above mentioned biological processes and pathways, the analysis results in this study provided potential presumptions of the molecular mechanism of ACP6 in HCC. Moreover, the in vitro experiments results in the present work supported the functional roles of ACP6 in proliferation and migration of HCC cells. The last high spot of this study was in vivo experiments on how NC affects the expression of ACP6 in HCC tissues. The pharmacologic mechanism of NC in tumor inhibition of HCC was far from been clarified and we conducted experiments on nude mice for further exploration. The significant suppression of NC on ACP6 in HCC tissues found in the present work might serve as a supplement to the explanations of pharmacologic actions of NC in fighting HCC.

Limitations of this paper were also existed. The protein expression of ACP6 in NC-treated xenograft and in Huh7 cells transfected with ACP6 siRNA should be examined by western blotting or immunohistochemistry. In vivo experiments are warranted in future work for validating the oncogenic roles of ACP6 in HCC and the molecular interactions between ACP6 and its co-expressed genes. The number of mice in the NC-treated was only two and control group was only three in the present study; both numbers of mice were too small for statistical analysis. The sufficient number of mice for subsequent statistical analysis should be eight to ten. More mice will be added in future in vivo experiments for guaranteeing the robustness of the in vivo experiment results.

## Conclusions

In summary, we proved the overexpression of ACP6 in HCC and promotive effect of up-regulated ACP6 in the aggressive development of HCC. ACP6 was one of the potential targets of NC in HCC. The findings in the present study was anticipated to shed light on the molecular mechanism of HCC and lay a theoretical foundation for application prospect of ACP6 as a biomarker for HCC.

## Supplementary Information

**Additional file 1:** **Figure 1. **Flowchart of the selection process of eligible RNA-seq datasets or microarrays for expression analysis.

**Additional file 2:** **Figure 2.** The expression pattern of ACP6 in HCC and non-cancer liver samples. Differential expression of ACP6 between HCC (marked in red) and non-cancer liver samples (marked in blue) was displayed in a panel of violin plot. N: non-cancer liver samples. T: HCC samples.

**Additional file 3:** **Figure 1. **Flowchart of the selection process of eligible RNA-seq datasets or microarrays for expression analysis.

**Additional file 4:** **Figure 4.** The overall expression trend of ACP6 in HCC and its discriminatory capacity. A. Forest plot of SMD. SMD: standard mean difference; SD: standard deviation. B. SROC curves. AUC: area under curve. SENS: sensitivity; SPEC: specificity.

**Additional file 5:** **Figure 5.** The associations between ACP6 expression and clinico-pathological variables of HCC patients. The violin plots showed ACP6 expression in HCC patients with different groups of adjacent hepatic tissue inflammation (A), history of hepatistis B (B), Ishak fibrosis scores (C) and histologic grades (D).

**Additional file 6:** **Figure 6.** Prognostic value of ACP6 expression for HCC patients. Kaplan-Meier survival curves were created based on prognostic data of HCC patients in E-TABM-36 (A), GSE76427 (B) and TCGA database (C). The forest plot of HR value summarized the overall effect of ACP6 expression on overall survival of HCC patients (D). HR: hazard ratio.

**Additional file 7:** **Figure 7.** Genetic alteration profile of ACP6 in HCC samples. HCC cases with genetic alterations of ACP6 were marked in different colors. GISTIC: genomic identification of significant targets in cancer.

**Additional file 8:** **Figure 8.** Functional annotations for genes co-expressed with ACP6. A. Network of enriched biological process or pathway terms colored by ID. B. Network of enriched biological process or pathway terms colored by p value.

**Additional file 9.**

**Additional file 10:** **Supplementary Table 1.** Detailed information of all included RNA-seq dataset and microarrays for the current work.

## Data Availability

The datasets supporting the conclusions of this article are available in TCGA (https://portal.gdc.cancer.gov/) (TCGA-LIHC), GEO (GSE31370, GSE36376, GSE36411, GSE39791, GSE57957, GSE76427, GSE114564, GSE148355, GSE63863, GSE65485, GSE73708, GSE81550, GSE87592, GSE104310, GSE63018, GSE77509, GSE94660, GSE97214, GSE140845, GSE112221, GSE101685, GSE102079, GSE107170, GSE112790, GSE121248, GSE17548, GSE19665, GSE29721, GSE33006, GSE41804, GSE45436, GSE6222, GSE62232, GSE6764, GSE99807, GSE84402, GSE14323, GSE14520, GSE17967, GSE9839, GSE57727, GSE98617, GSE113996, GSE74656, GSE101728, GSE98269, GSE12941, GSE84005, GSE45050, GSE64041, GSE117361, GSE54236, GSE87630, GSE89377, GSE25599, GSE77314, GSE67764, GSE60502, GSE124535, GSE22405, GSE25097, GSE33294, GSE46444, GSE63898, GSE50579, GSE56545, GSE57555, GSE54238, GSE76311, GSE20140, GSE115018, GSE125469, GSE166163, GSE46408, GSE22058, GSE55048, GSE59259, GSE114783) (https://www.ncbi.nlm.nih.gov/gds) and ArrayExpress (E-MTAB-8887, E-MTAB-4171) (https://www.ebi.ac.uk/arrayexpress/) databases. RNA-seq data of HCC xenograft tissues treated with or without nitidine chloride was uploaded to Mendeley data (https://data.mendeley.com/datasets/stkrjf7w7t/1). Raw data of in vitro experiments from the current study was available in FigShare (https://figshare.com/articles/dataset/Raw_data_of_in_vitro_experiments/20337021). Public access to all above databases is open.

## Abbreviations

ACP6: Acid phosphatase type 6
HCC: Hepatocellular carcinoma
SMD: Standard mean difference
AUC: Area under curve
NC: Nitidine chloride
HBV: Hepatitis B virus
FPKM: Fragments per kilobase million
TPM: Transcripts per million
TCGA: The cancer genome atlas
GTEx: Genotype-tissue expression project
GEO: Gene expression omnibus
ROC: Receiver’s operating characteristics
SROC: Summarized receiver’s operating characteristics
CI: Confidence interval
HR: Hazard ratio
tNSE: T-distributed stochastic neighbor embedding
MEGENA: Multiscale embedded gene co-expression network analysis
SD: Standard deviation
M: Mean
