# Supplementary material for: The expression characteristics and clinical significance of ACP6, a potential target of nitidine chloride, in hepatocellular carcinoma
Source: BMC Cancer. 2022 Dec 1;22:1244. doi: 10.1186/s12885-022-10292-1 (PMC9714191; doi:10.1186/s12885-022-10292-1)

**Additional figure 1.** Flowchart of the selection process of eligible RNA-seq datasets or microarrays for expression analysis.


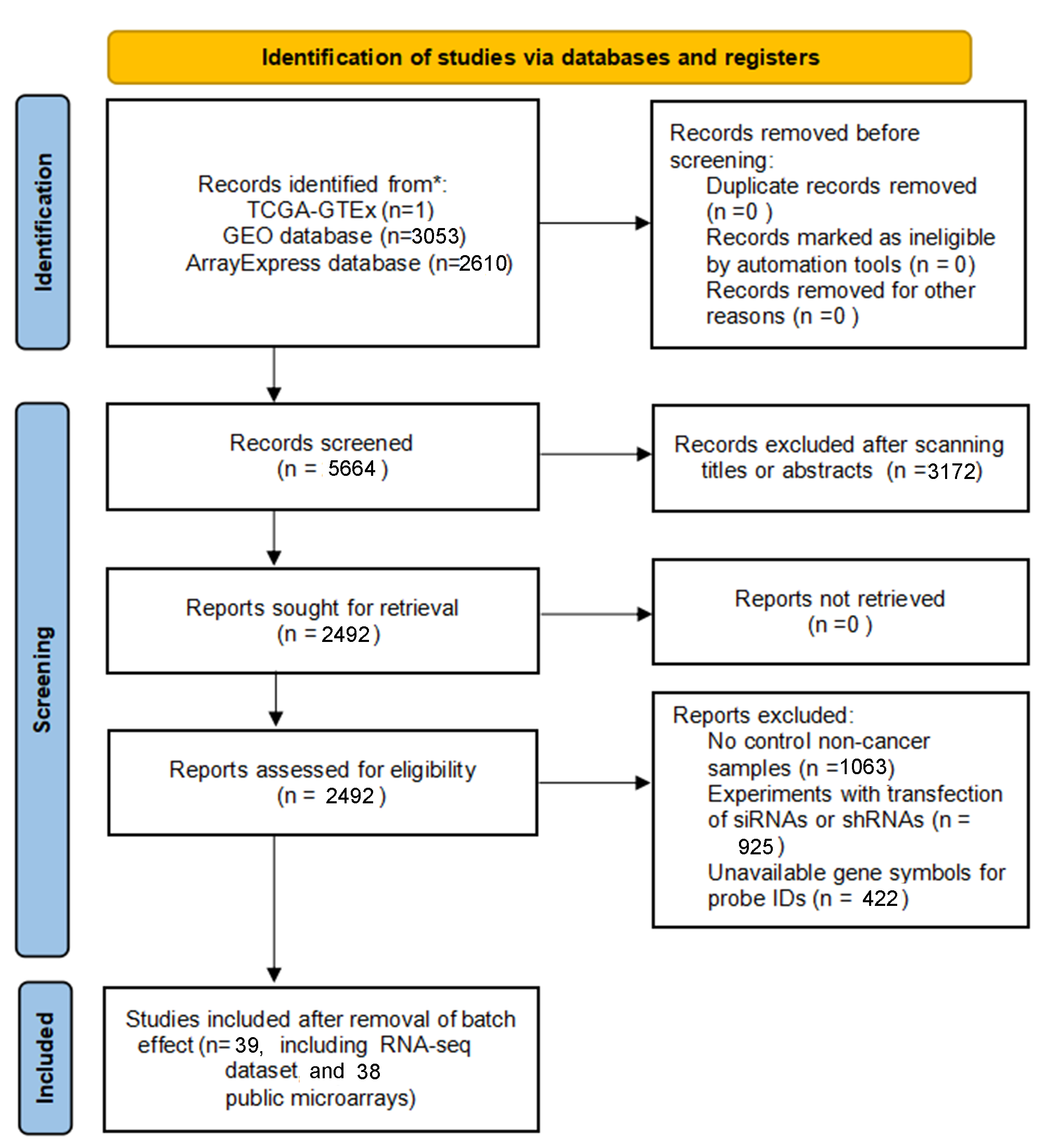

Supplement: Supplementary file 1 — Additional file 1: Figure 1. Flowchart of the selection process of eligible RNA-seq datasets or microarrays for expression analysis. [file 12885_2022_10292_MOESM1_ESM.docx]
