# Supplementary material for: The expression characteristics and clinical significance of ACP6, a potential target of nitidine chloride, in hepatocellular carcinoma
Source: BMC Cancer. 2022 Dec 1;22:1244. doi: 10.1186/s12885-022-10292-1 (PMC9714191; doi:10.1186/s12885-022-10292-1)
Supplement: Supplementary file 2 — Additional file 2: Figure 2. The expression pattern of ACP6 in HCC and non-cancer liver samples. Differential expression of ACP6 between HCC (marked in red) and non-cancer liver samples (marked in blue) was displayed in a panel of violin plot. N: non-cancer liver samples. T: HCC samples. [file 12885_2022_10292_MOESM2_ESM.docx]

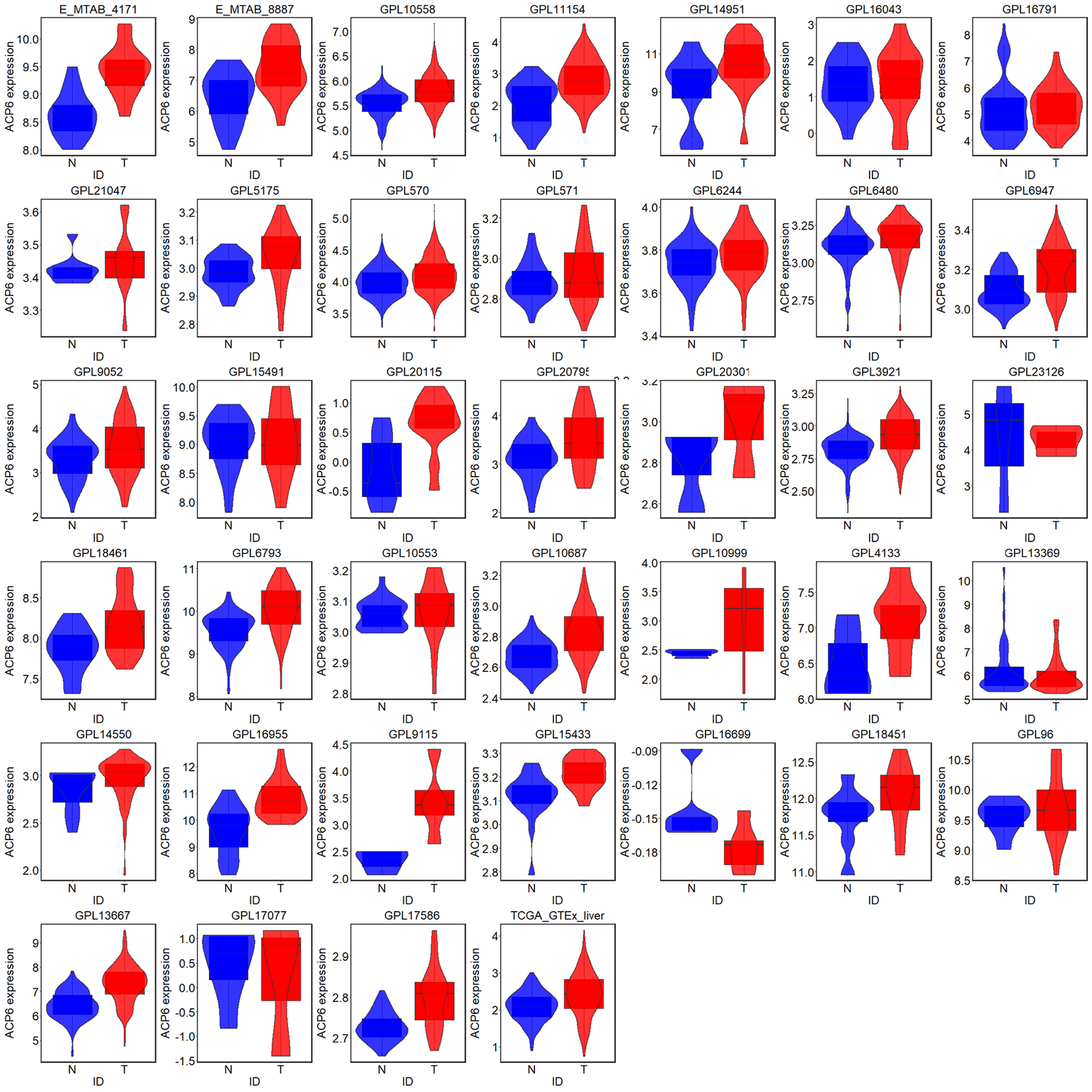
**Additional figure 2.** The expression pattern of ACP6 in HCC and non-cancer liver samples. Differential expression of ACP6 between HCC (marked in red) and non-cancer liver samples (marked in blue) was displayed in a panel of violin plot. N: non-cancer liver samples. T: HCC samples.

10292
