# Supplementary material for: The expression characteristics and clinical significance of ACP6, a potential target of nitidine chloride, in hepatocellular carcinoma
Source: BMC Cancer. 2022 Dec 1;22:1244. doi: 10.1186/s12885-022-10292-1 (PMC9714191; doi:10.1186/s12885-022-10292-1)

**Additional figure 1.** Flowchart of the selection process of eligible RNA-seq datasets or microarrays for expression analysis.


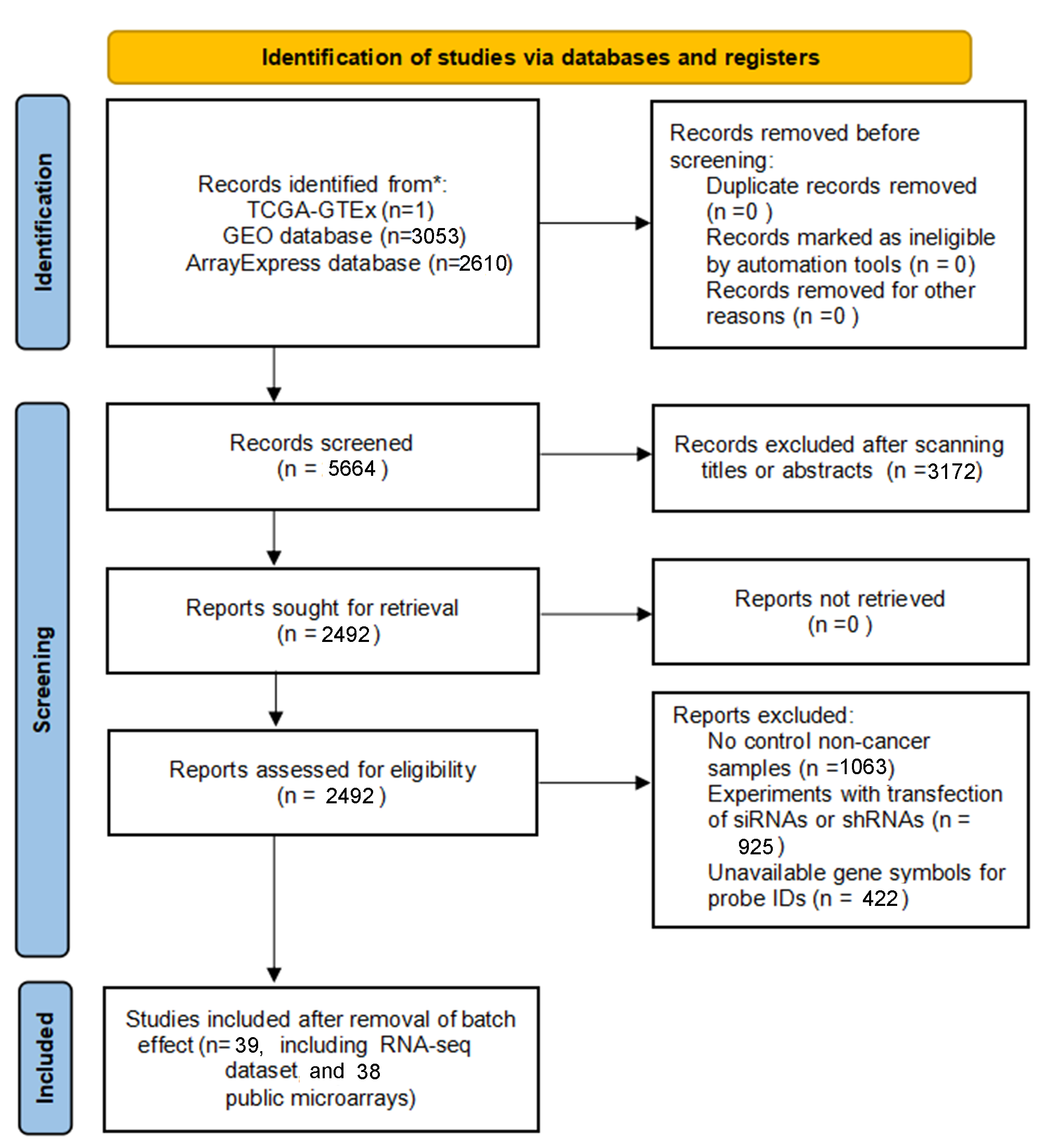


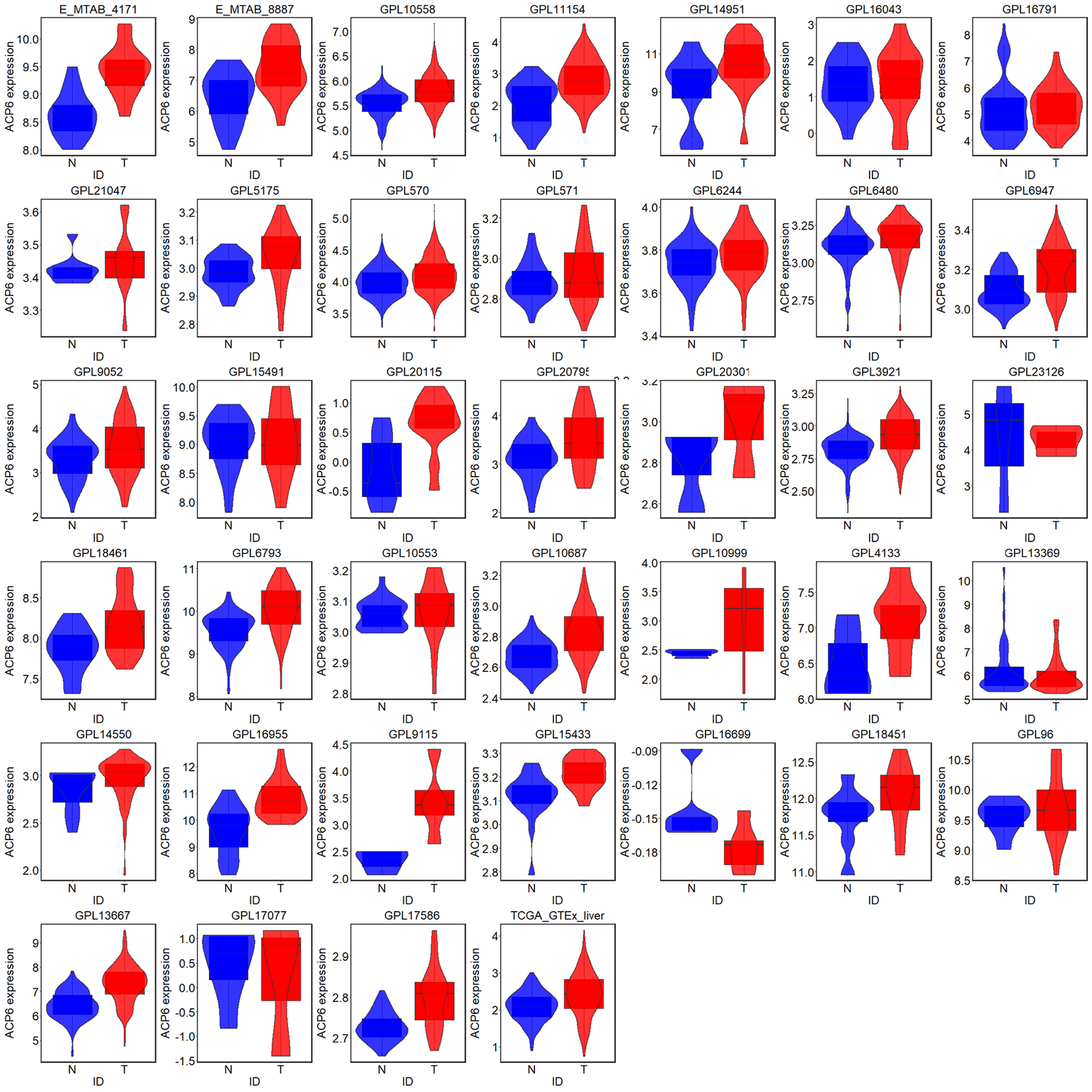
**Additional figure 2.** The expression pattern of ACP6 in HCC and non-cancer liver samples. Differential expression of ACP6 between HCC (marked in red) and non-cancer liver samples (marked in blue) was displayed in a panel of violin plot. N: non-cancer liver samples. T: HCC samples.


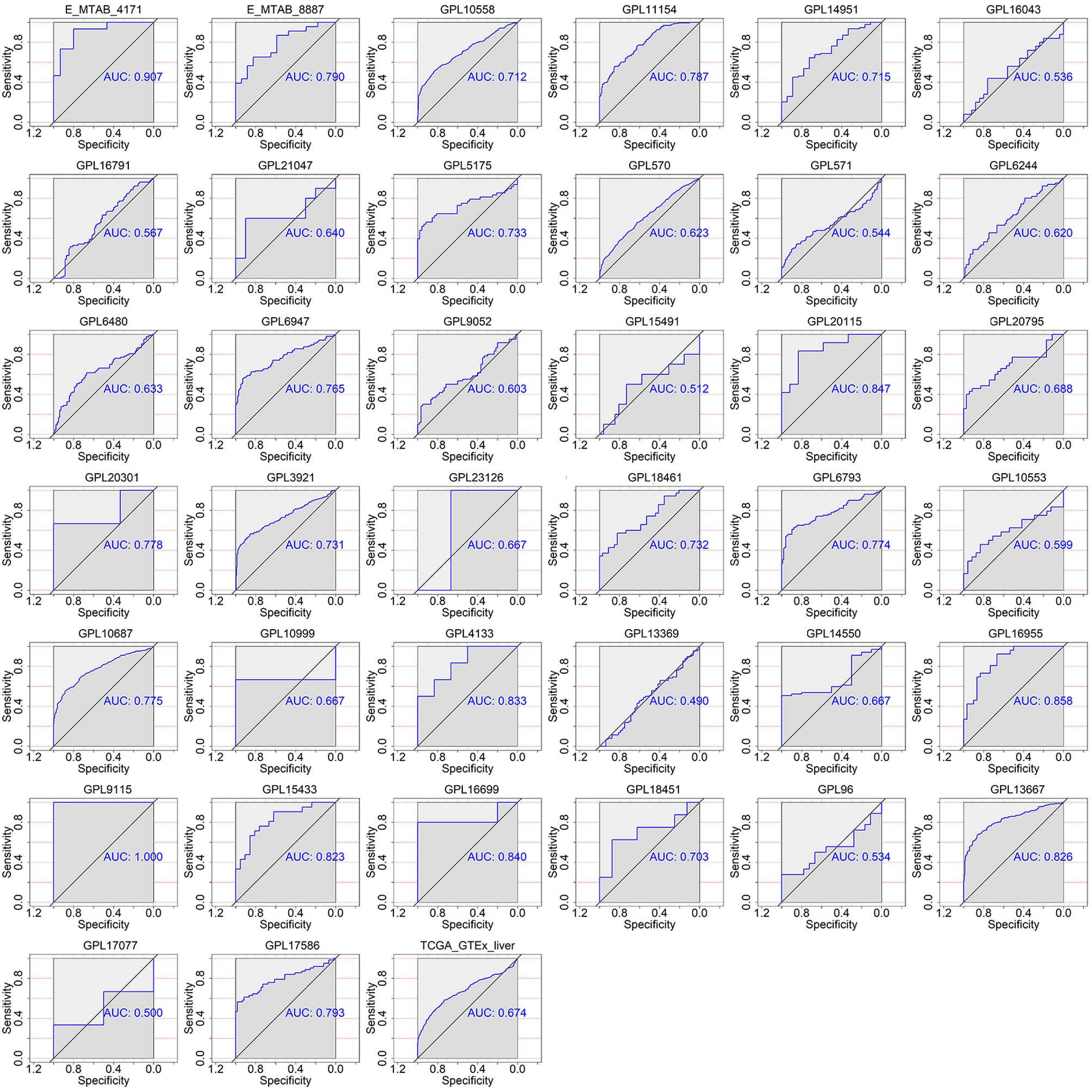
**Additional figure 3.** The distinguishing ability of ACP6 expression in screening HCC samples from non-cancer liver samples. AUC: area under curve.

**Additional figure 4.** The overall expression trend of ACP6 in HCC and its discriminatory capacity. A. Forest plot of SMD. SMD: standard mean difference; SD: standard deviation. B. SROC curves. AUC: area under curve. SENS: sensitivity; SPEC: specificity.


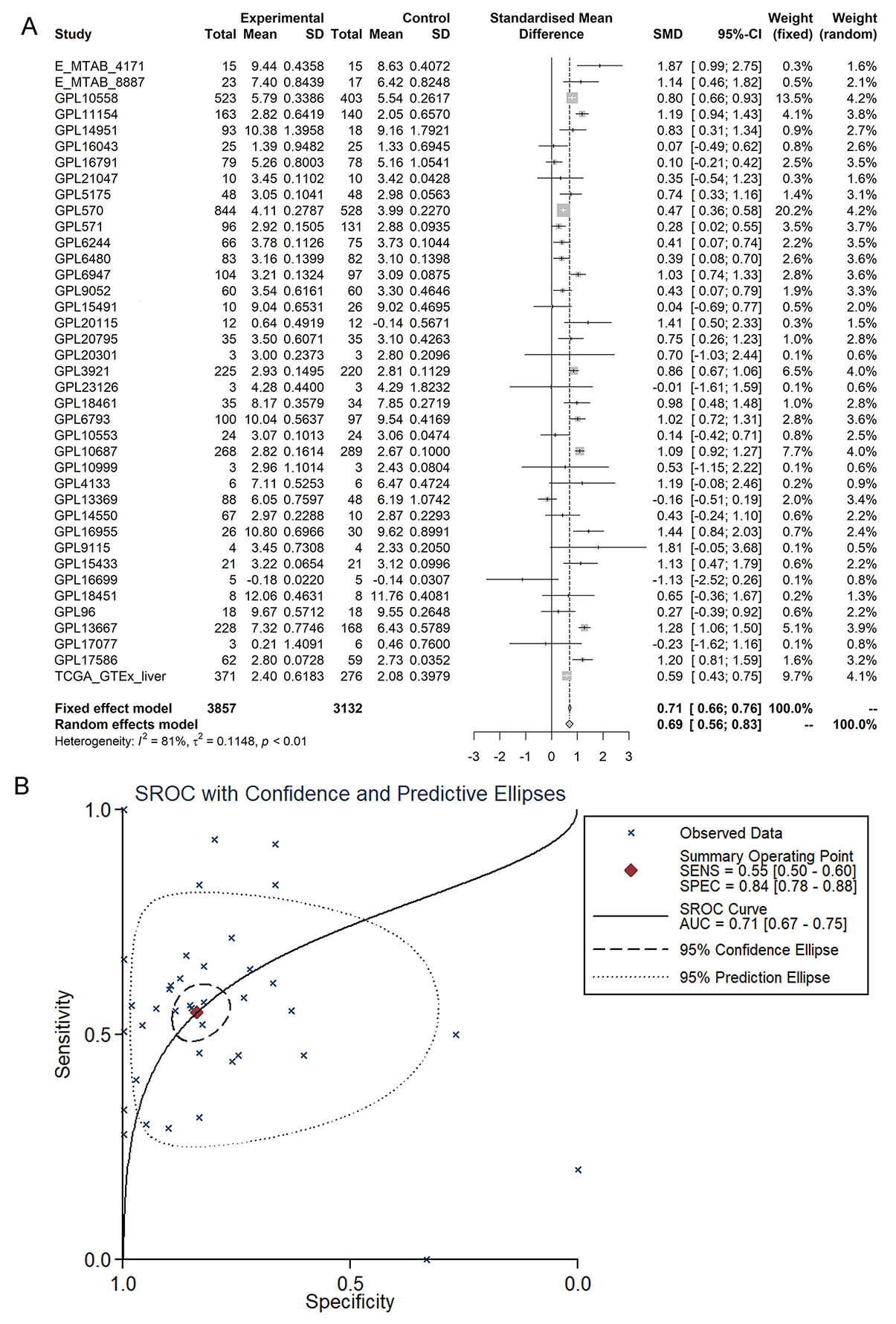


**Additional figure 5.** The associations between ACP6 expression and clinico-pathological variables of HCC patients. The violin plots showed ACP6 expression in HCC patients with different groups of adjacent hepatic tissue inflammation (A), history of hepatistis B (B), Ishak fibrosis scores (C) and histologic grades (D).


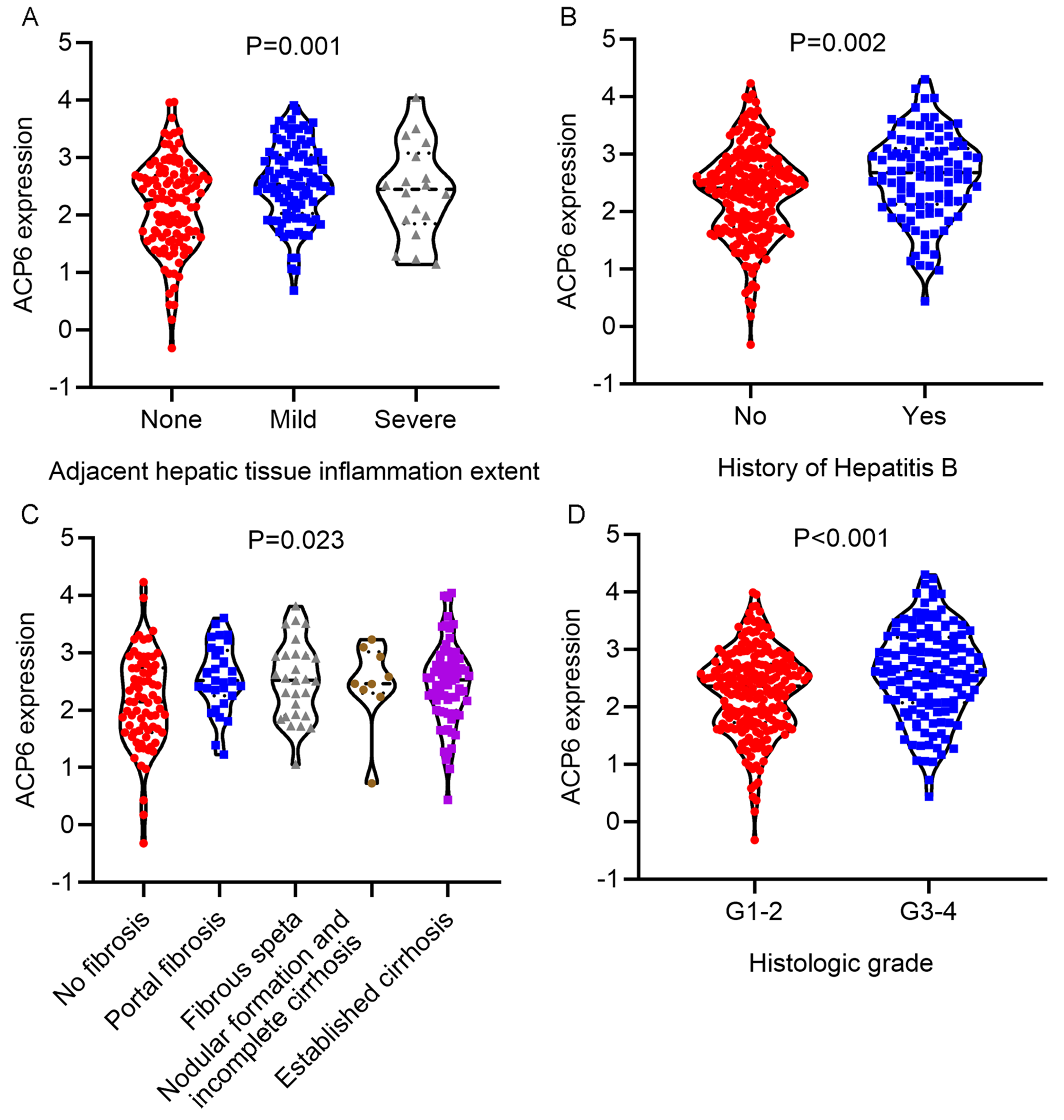


**Additional figure 6.** Prognostic value of ACP6 expression for HCC patients. Kaplan-Meier survival curves were created based on prognostic data of HCC patients in E-TABM-36 (A), GSE76427 (B) and TCGA database (C). The forest plot of HR value summarized the overall effect of ACP6 expression on overall survival of HCC patients (D). HR: hazard ratio.


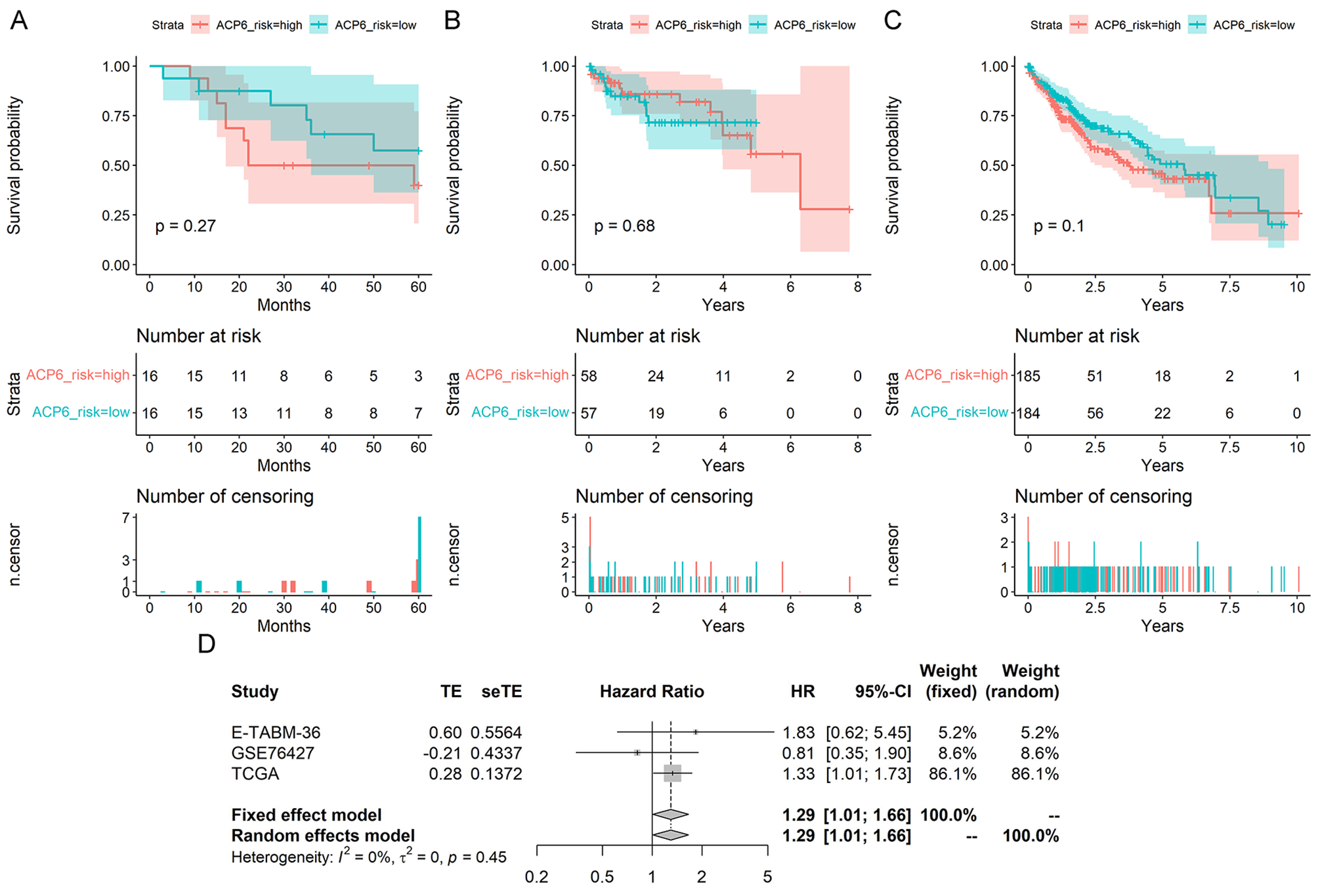


**Additional figure 7.** Genetic alteration profile of ACP6 in HCC samples. HCC cases with genetic alterations of ACP6 were marked in different colors. GISTIC: genomic identification of significant targets in cancer.


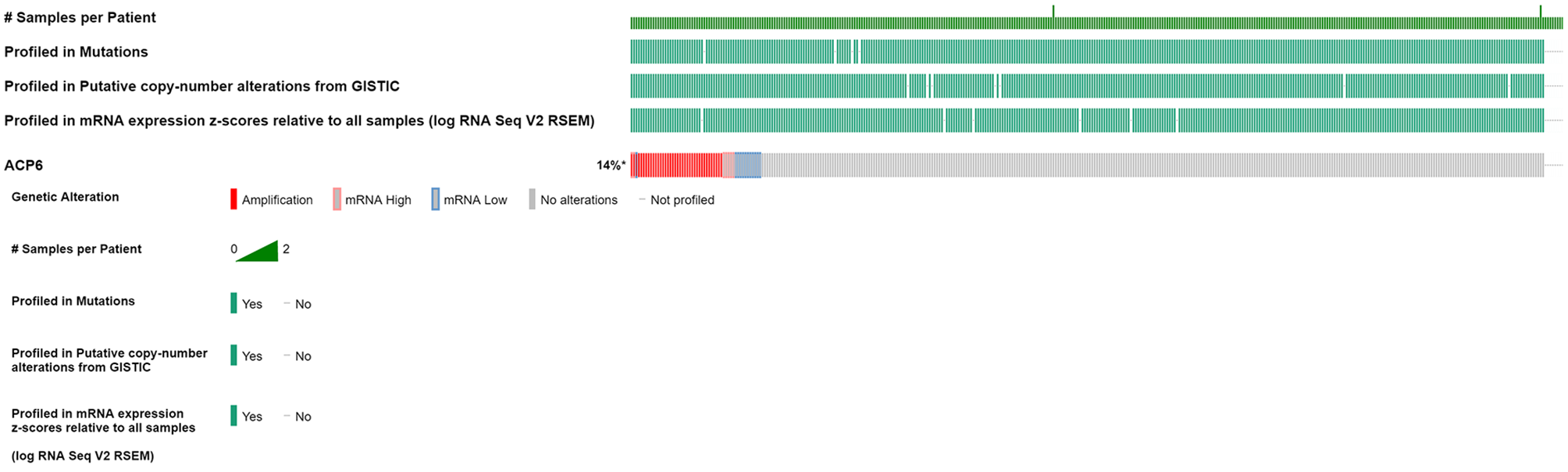


**Additional figure 8.** Functional annotations for genes co-expressed with ACP6. A. Network of enriched biological process or pathway terms colored by ID. B. Network of enriched biological process or pathway terms colored by p value.


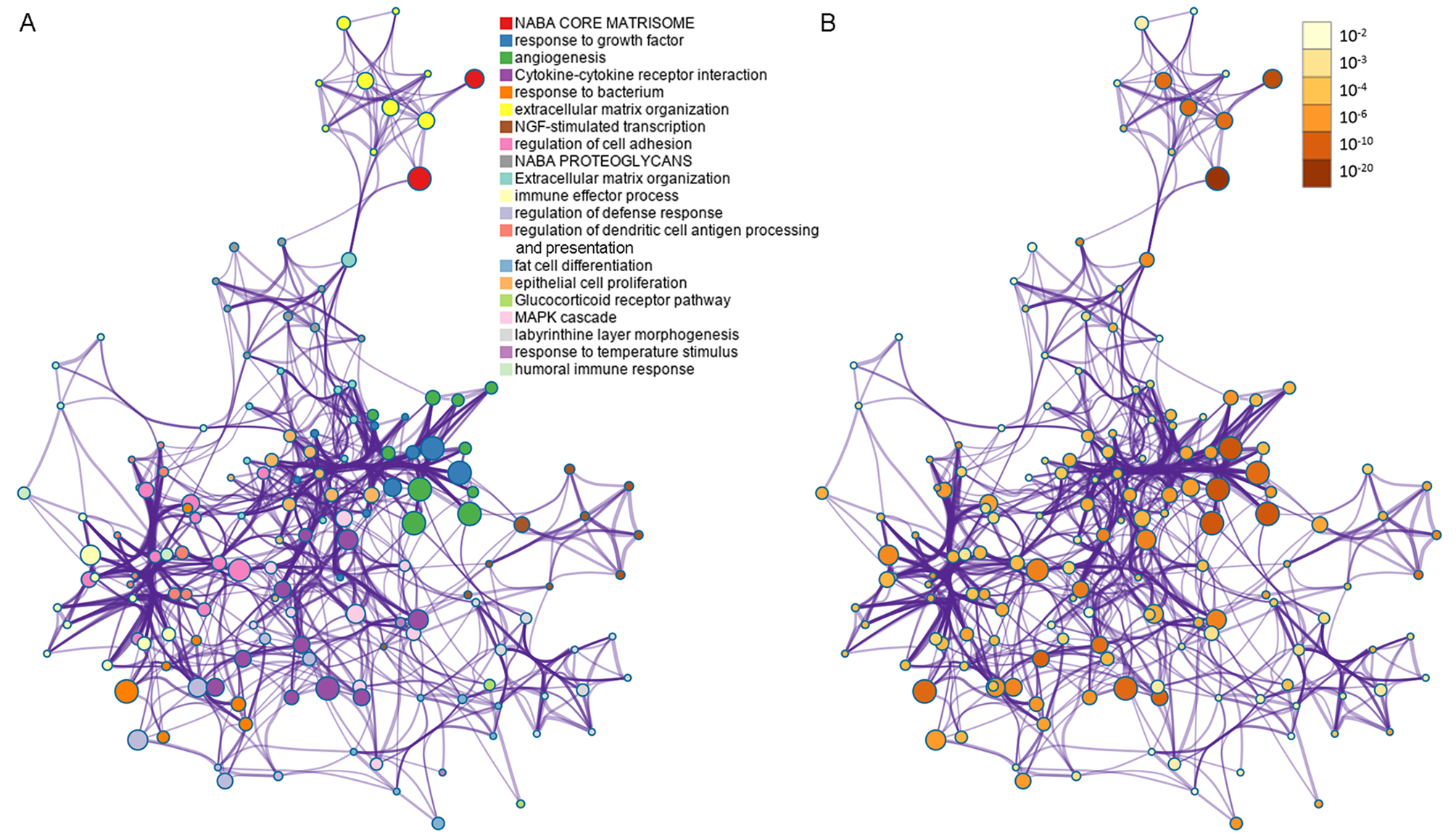

Supplement: Supplementary file 3 — Additional file 3: Figure 1. Flowchart of the selection process of eligible RNA-seq datasets or microarrays for expression analysis. [file 12885_2022_10292_MOESM3_ESM.docx]
