# Supplementary material for: The expression characteristics and clinical significance of ACP6, a potential target of nitidine chloride, in hepatocellular carcinoma
Source: BMC Cancer. 2022 Dec 1;22:1244. doi: 10.1186/s12885-022-10292-1 (PMC9714191; doi:10.1186/s12885-022-10292-1)

**Additional figure 4.** The overall expression trend of ACP6 in HCC and its discriminatory capacity. A. Forest plot of SMD. SMD: standard mean difference; SD: standard deviation. B. SROC curves. AUC: area under curve. SENS: sensitivity; SPEC: specificity.


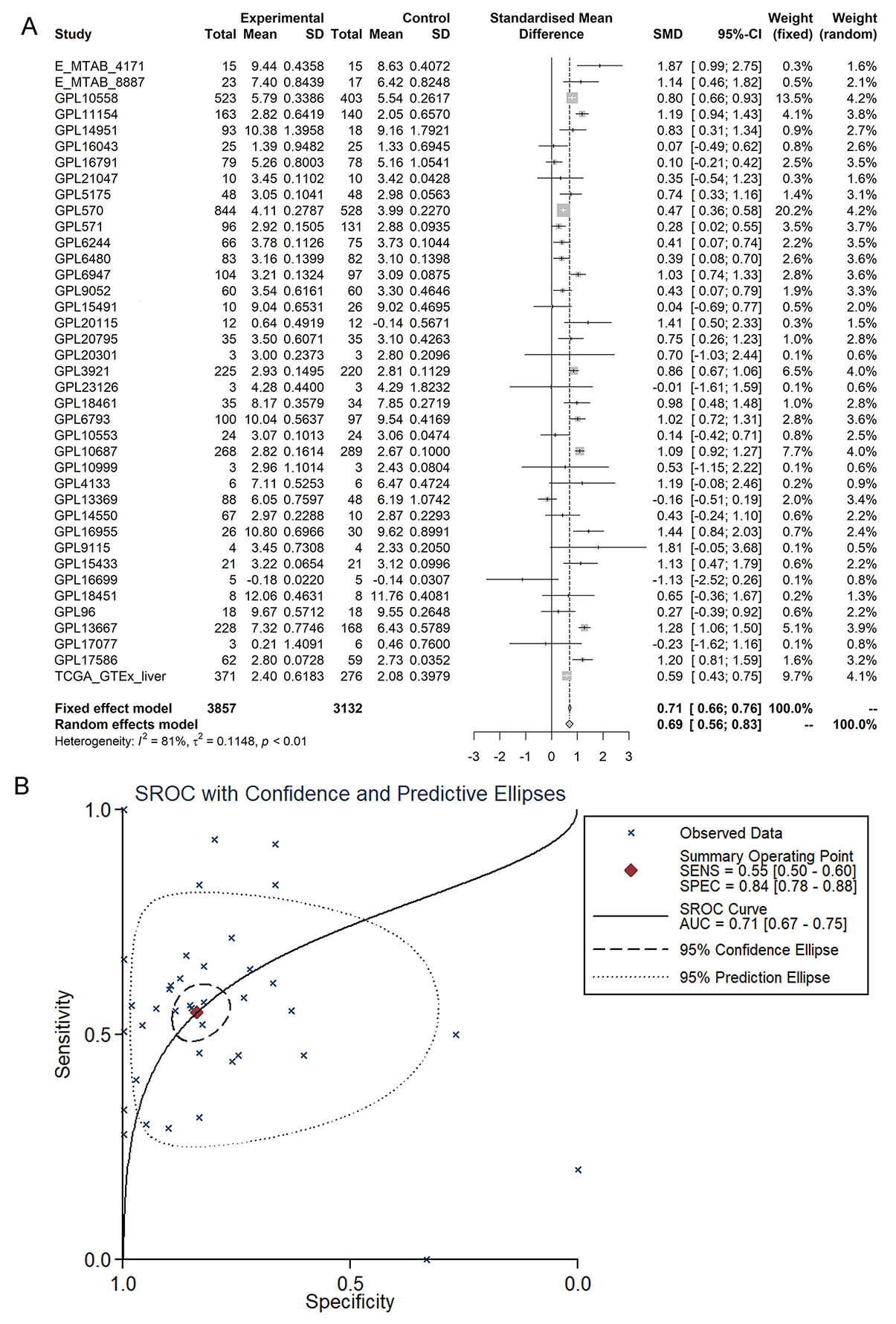

Supplement: Supplementary file 4 — Additional file 4: Figure 4. The overall expression trend of ACP6 in HCC and its discriminatory capacity. A. Forest plot of SMD. SMD: standard mean difference; SD: standard deviation. B. SROC curves. AUC: area under curve. SENS: sensitivity; SPEC: specificity. [file 12885_2022_10292_MOESM4_ESM.docx]
