# Supplementary material for: The expression characteristics and clinical significance of ACP6, a potential target of nitidine chloride, in hepatocellular carcinoma
Source: BMC Cancer. 2022 Dec 1;22:1244. doi: 10.1186/s12885-022-10292-1 (PMC9714191; doi:10.1186/s12885-022-10292-1)

**Additional figure 5.** The associations between ACP6 expression and clinico-pathological variables of HCC patients. The violin plots showed ACP6 expression in HCC patients with different groups of adjacent hepatic tissue inflammation (A), history of hepatistis B (B), Ishak fibrosis scores (C) and histologic grades (D).


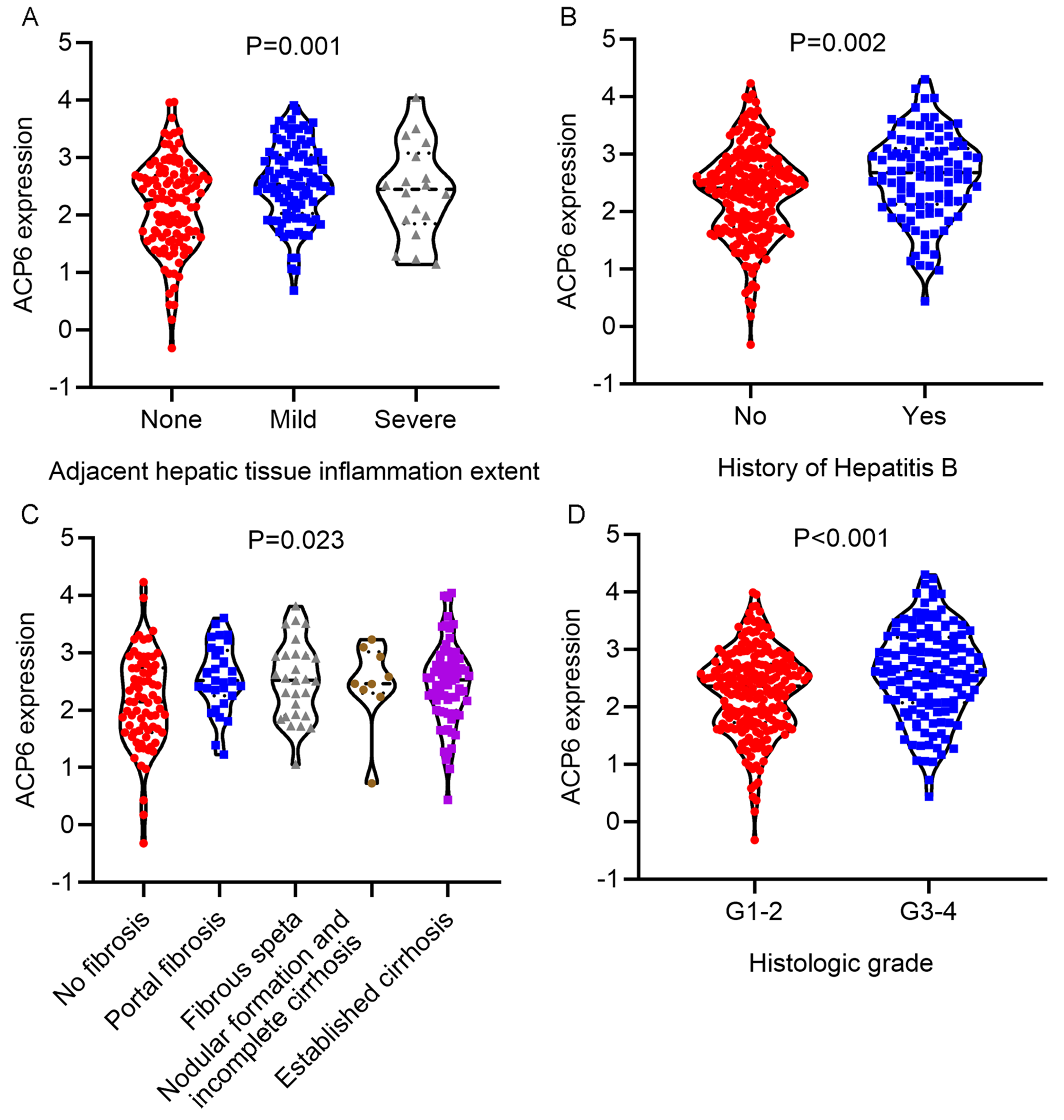

Supplement: Supplementary file 5 — Additional file 5: Figure 5. The associations between ACP6 expression and clinico-pathological variables of HCC patients. The violin plots showed ACP6 expression in HCC patients with different groups of adjacent hepatic tissue inflammation (A), history of hepatistis B (B), Ishak fibrosis scores (C) and histologic grades (D). [file 12885_2022_10292_MOESM5_ESM.docx]
