# Supplementary material for: The expression characteristics and clinical significance of ACP6, a potential target of nitidine chloride, in hepatocellular carcinoma
Source: BMC Cancer. 2022 Dec 1;22:1244. doi: 10.1186/s12885-022-10292-1 (PMC9714191; doi:10.1186/s12885-022-10292-1)

**Additional figure 6.** Prognostic value of ACP6 expression for HCC patients. Kaplan-Meier survival curves were created based on prognostic data of HCC patients in E-TABM-36 (A), GSE76427 (B) and TCGA database (C). The forest plot of HR value summarized the overall effect of ACP6 expression on overall survival of HCC patients (D). HR: hazard ratio.


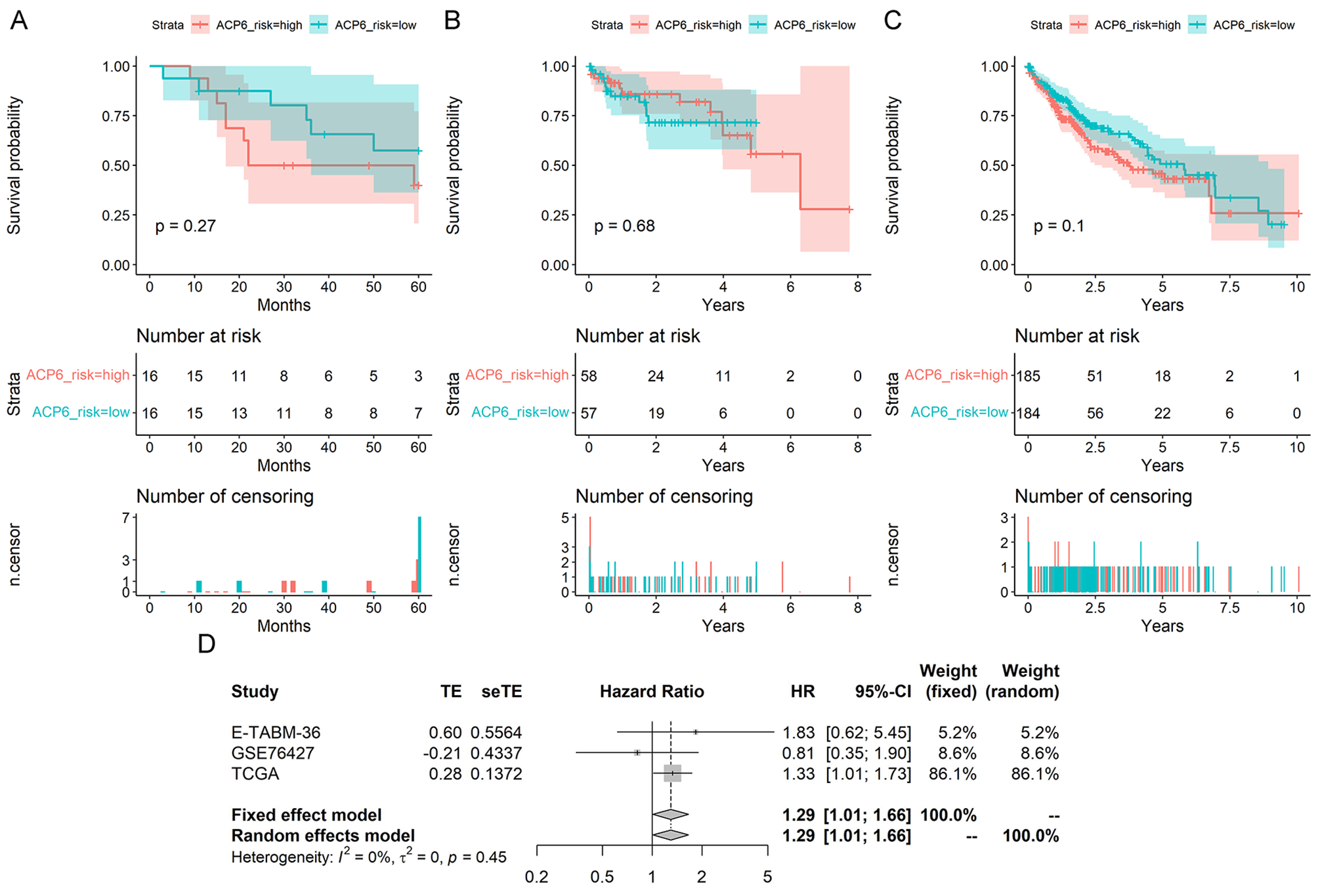

Supplement: Supplementary file 6 — Additional file 6: Figure 6. Prognostic value of ACP6 expression for HCC patients. Kaplan-Meier survival curves were created based on prognostic data of HCC patients in E-TABM-36 (A), GSE76427 (B) and TCGA database (C). The forest plot of HR value summarized the overall effect of ACP6 expression on overall survival of HCC patients (D). HR: hazard ratio. [file 12885_2022_10292_MOESM6_ESM.docx]
