# Supplementary material for: The expression characteristics and clinical significance of ACP6, a potential target of nitidine chloride, in hepatocellular carcinoma
Source: BMC Cancer. 2022 Dec 1;22:1244. doi: 10.1186/s12885-022-10292-1 (PMC9714191; doi:10.1186/s12885-022-10292-1)

**Additional figure 7.** Genetic alteration profile of ACP6 in HCC samples. HCC cases with genetic alterations of ACP6 were marked in different colors. GISTIC: genomic identification of significant targets in cancer.


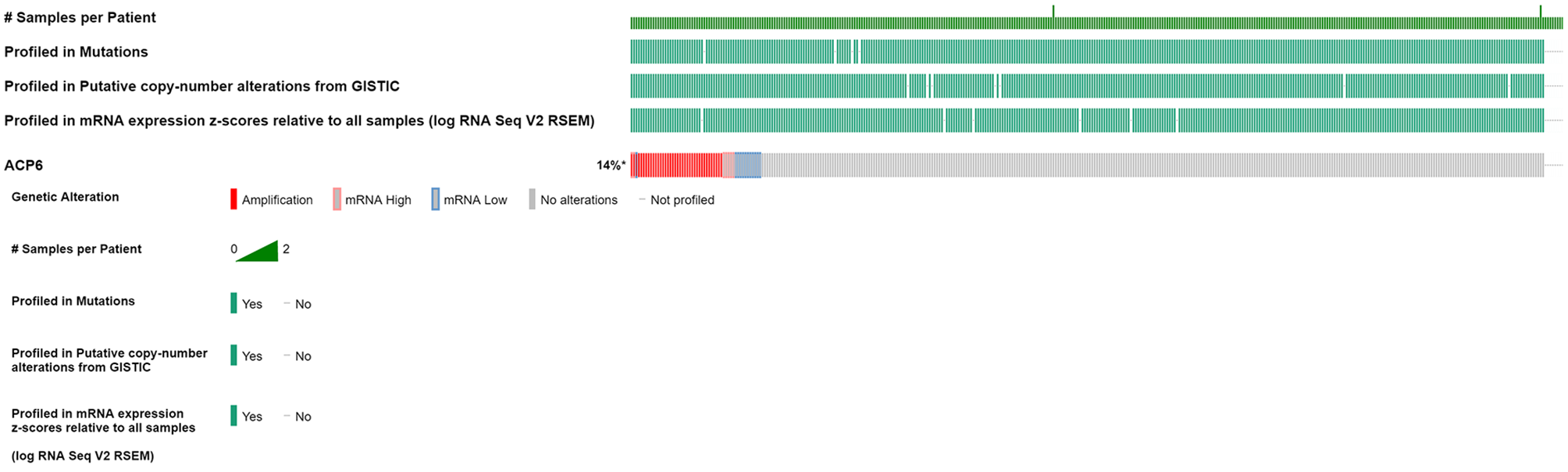

Supplement: Supplementary file 7 — Additional file 7: Figure 7. Genetic alteration profile of ACP6 in HCC samples. HCC cases with genetic alterations of ACP6 were marked in different colors. GISTIC: genomic identification of significant targets in cancer. [file 12885_2022_10292_MOESM7_ESM.docx]
