# Supplementary material for: The expression characteristics and clinical significance of ACP6, a potential target of nitidine chloride, in hepatocellular carcinoma
Source: BMC Cancer. 2022 Dec 1;22:1244. doi: 10.1186/s12885-022-10292-1 (PMC9714191; doi:10.1186/s12885-022-10292-1)

**Additional figure 8.** Functional annotations for genes co-expressed with ACP6. A. Network of enriched biological process or pathway terms colored by ID. B. Network of enriched biological process or pathway terms colored by p value.


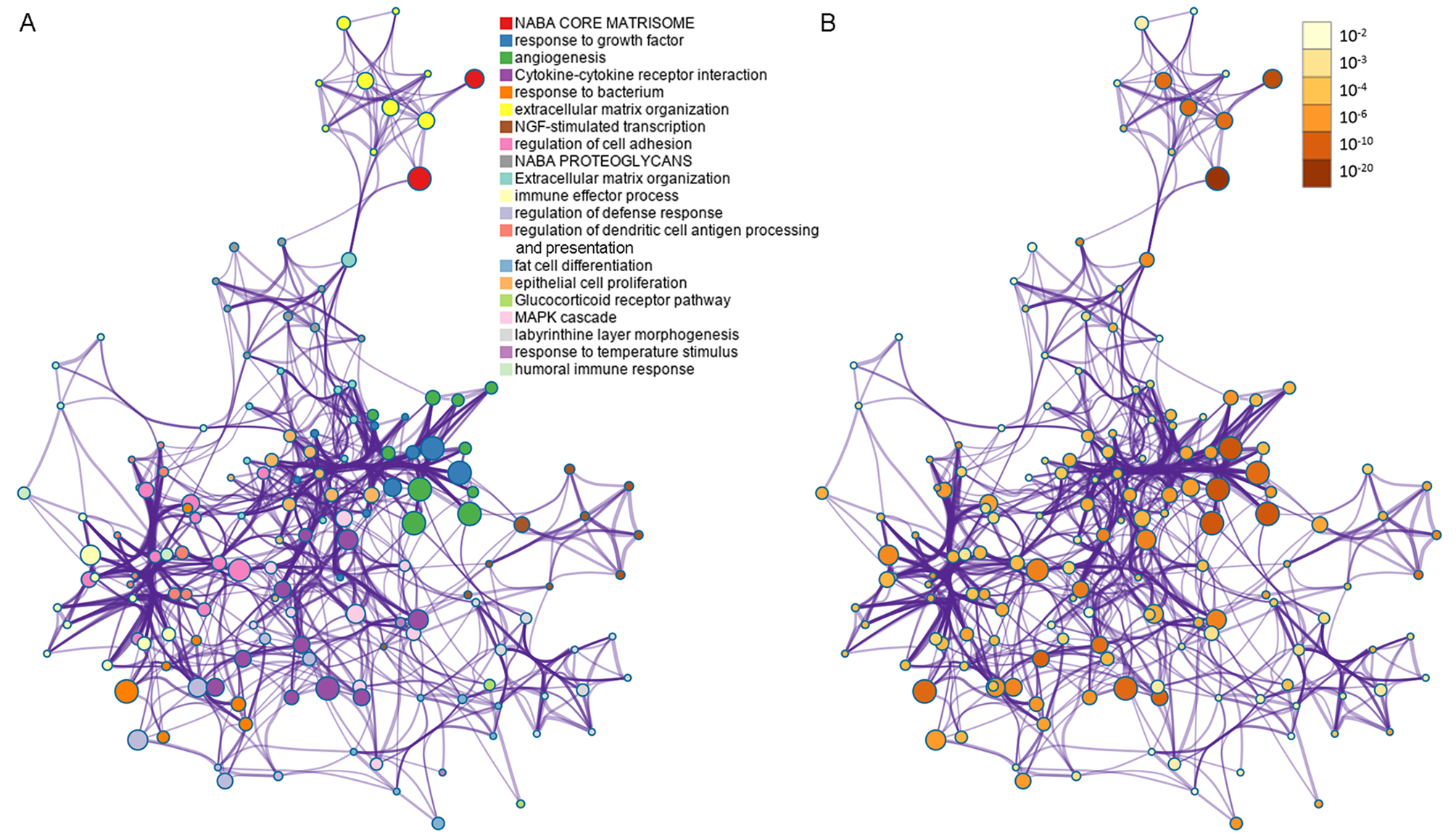

Supplement: Supplementary file 8 — Additional file 8: Figure 8. Functional annotations for genes co-expressed with ACP6. A. Network of enriched biological process or pathway terms colored by ID. B. Network of enriched biological process or pathway terms colored by p value. [file 12885_2022_10292_MOESM8_ESM.docx]
